# Harnessing the power of machine learning for crop improvement and sustainable production

**DOI:** 10.3389/fpls.2024.1417912

**Published:** 2024-08-12

**Authors:** Seyed Mahdi Hosseiniyan Khatibi, Jauhar Ali

**Affiliations:** Rice Breeding Platform, International Rice Research Institute, Los Baños, Laguna, Philippines

**Keywords:** artificial intelligence, machine learning, deep learning, precision crop improvement, prediction model

## Abstract

Crop improvement and production domains encounter large amounts of expanding data with multi-layer complexity that forces researchers to use machine-learning approaches to establish predictive and informative models to understand the sophisticated mechanisms underlying these processes. All machine-learning approaches aim to fit models to target data; nevertheless, it should be noted that a wide range of specialized methods might initially appear confusing. The principal objective of this study is to offer researchers an explicit introduction to some of the essential machine-learning approaches and their applications, comprising the most modern and utilized methods that have gained widespread adoption in crop improvement or similar domains. This article explicitly explains how different machine-learning methods could be applied for given agricultural data, highlights newly emerging techniques for machine-learning users, and lays out technical strategies for agri/crop research practitioners and researchers.

## 1 Introduction

Naturally, humans’ learning procedure is carefully or randomly monitoring surrounding events, grasping some experience and then predicting the next event, mainly occurring without human awareness. For instance, consider a human child who is learning how to talk. Basically, children do not know language learning techniques, procedures, or linguistics. Nevertheless, by listening to surrounding sounds, experimenting, and making mistakes, children gradually adjust their listening skills and simultaneously learn to talk and communicate in different situations. These procedures will continue until children feel confident enough to speak. Technically, they are learning how to talk by establishing a sound and an adequately accurate model of a whole set of procedures automatically and by testing the developed model again and again with surrounding voice data and improving it to build a more precise model. The term “machine learning” typically refers to the procedures of finding relevant groups within data or fitting prediction models to a target dataset. In essence, machine learning aims to mimic or resemble human capacity and the ability to identify patterns using computation approaches. Machine learning is especially handy when the dataset being analyzed is huge or sophisticated beyond human ability to analyze it or when we want to build an automated platform for analyzing a target dataset by considering it to be time-efficient and repeatable. Agricultural data often have these characteristics. Over the past few decades, agricultural databases have experienced remarkable growth in quantity and multi-layer complexity. Having a solid grasp of the methods being employed and some valuable tools for interpreting this wealth of data is becoming increasingly crucial. Although machine learning has been engaged in the crop domain for many years, its usage in agriculture and crop improvement has now become so widespread that it is used in almost every discipline. Only recently, though, has the field started to examine the various strategies more closely to determine which ones work best in certain situations or whether they are suitable at all. This review aims to offer compact, sufficient, and explicit information and details on how to use machine-learning techniques for agricultural and crop improvement researchers. We do not seek to provide a comprehensive analysis and investigate the literature on machine-learning applications for crop improvement problems nor to get into the specific mathematical details of different machine-learning techniques (Liakos et al., 2018; Sharma et al., 2020). We focus on connecting specific machine-learning methods to various kinds of agricultural data. In addition, we will try to explain some best practices for approaching training and modeling improvement in real-world scenarios. The intrinsic intricacy of agricultural data poses opportunities and challenges for analytical methods in machine learning. We highlight common problems that undermine the validity of research and offer advice on how to overcome these challenges. The discussion of several machine-learning methods takes up most of this review, and we also provide explicit examples of how to use the strategy appropriately and understand the outcomes in each case. Traditional machine-learning techniques are included in the discussion as, in many situations; they continue to be the best options to apply. Our discussion covers techniques of deep learning, which shows satisfactory performance and is the best option for various machine-learning responsibilities. We also cover federated learning as a robust technique for having a machine-learning global crop improvement model to deal with future challenges such as climate change. We conclude by outlining the prospects for integrating machine learning into agricultural data analysis pipelines. When using machine learning in agriculture, there are two primary objectives. First, even though the collected data are sufficient or deficient, precise predictions should be made and used to direct further research endeavors. Since scientists are interested in understanding mechanisms, the other objective is to apply machine learning to enhance and increase the comprehension of crop improvement mechanisms, including several types of phenotypical, genotypical, biological, agronomic, and climatic mechanisms. We also summarize some of the limitations and applications of machine-learning approaches along with some data-related concerns for researchers in the crop improvement domain.

## 2 Shortlist of machine-learning applications for crop improvement and production

**Figure 1:**
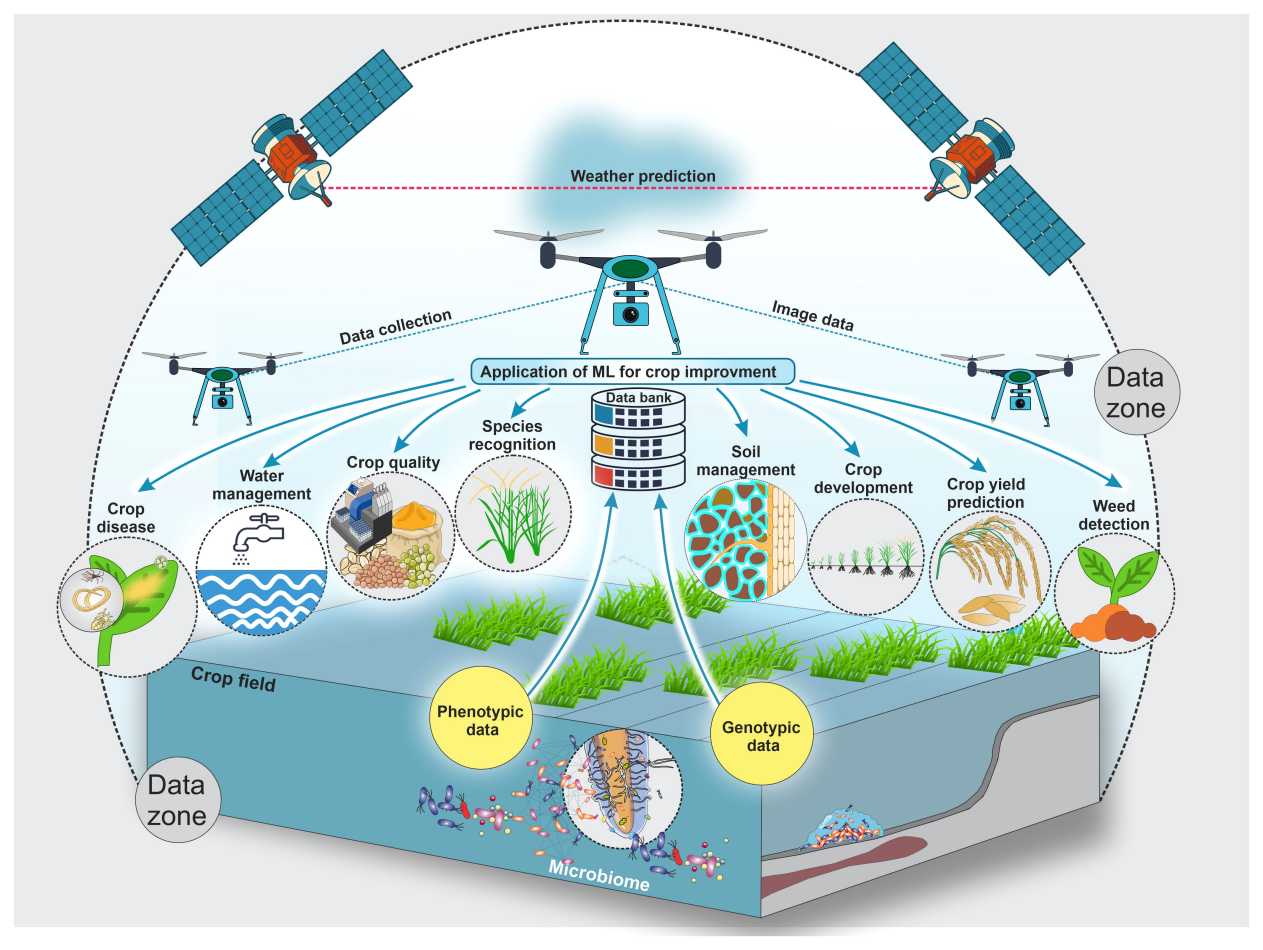
This schematic illustrates key applications of artificial intelligence and machine learning for crop development and improvement, including crop diseases, crop quality, crop species recognition, crop development, crop yield prediction, crop-related microbiome improvement, water management, soil management, etc. Farmers and researchers still encounter numerous obstacles due to employing traditional methods in the crop sector. Artificial intelligence and machine learning are used extensively to address these issues. Also, this figure shows possible data types and collection zones from crop fields to feed different machine-learning models to improve and develop different crops.

With emerging new technologies and approaches, large datasets are generated from different agricultural domains, particularly from the crop production domain. These vast datasets can easily feed into machine-learning approaches to help all beneficiaries optimize crop improvement systems. Even though machine-learning applications are extensive, their subcategories, mainly in crop quality (Elbasi et al., 2023; Attri et al., 2024), crop phenotyping (Gano et al., 2024), crop weed identification (Hu et al., 2021; Modi et al., 2023; Venkataraju et al., 2023), disease detection (Kulkarni and Shastri, 2024; Srinivas et al., 2024), crop recognition (Tian et al., 2021; Fu et al., 2023; Gafurov et al., 2023), crop-related microbiome improvements (Chang et al., 2017; Aguilar-Zambrano et al., 2023), and yield prediction (Van Klompenburg et al., 2020; Morales and Villalobos, 2023), were separated into crop development, production, and improvement, as shown in **Figure 1**.

## 3 Essential concepts

We discuss several fundamental ideas in machine learning and, whenever possible, present examples from agricultural literature to clarify these concepts.

### 3.1 Basic terms in machine learning

A dataset consists of several instances, or data points, that are conceptualized as individual experimental observations. Several fixed features describe each data point. Phenotype, genotype (SNPs), product price, and climatic parameters are a few examples of these features. Whatever we aim to do with a machine-learning model is specified objectively by a machine-learning task. For instance, we could predict the rate of price fluctuation at a particular point in time for a specific agricultural product with an experiment examining the cost of the crop product over time. In this instance, the features “cost of crop product” and “time” could be referred to as input features. The conversion rate, which would represent the anticipated output of the target model at a specific moment, is the quantity we are interested in forecasting. Input and output features of a model can be as many as desired. Features could be either categorical (accepting just discrete values) or continuous (continuous numerical values are used). Technically, categorical features are usually binary in nature, meaning they can be 1 (true) or 0 (false).

### 3.2 Concept of supervised, unsupervised, semi-supervised, and reinforcement learning

Supervised machine learning describes how a model can be fitted to data or part of target data that distinct labels have received for which a ground truth attribute exists; this quality is often determined by experimentation, researchers, or data collectors. In contrast to knowledge derived from inference, ground truth is information verified via direct observation and measurement, thus known to be accurate or real (Kondermann, 2013). Among the examples are high-yield prediction (Panigrahi et al., 2023) and water quality prediction (Ahmed et al., 2019; Ghosh et al., 2023; Chatterjee et al., 2024) using supervised learning for crop improvement. Laboratory or experimental observations ultimately serve as the source of ground truth in both cases. Contrary to supervised learning, patterns in unlabeled data can be found using unsupervised-learning techniques (James et al., 2023). This approach does not require predetermined labels with ground truth information (Sindhu Meena and Suriya, 2020). For example, plant image data can be analyzed using an unsupervised machine-learning technique (Davis et al., 2020; Bah et al., 2023). Semi-supervised learning, in which a significant quantity of unlabeled data is paired with tiny quantities of labeled data, occasionally combines the two methodologies (Ouali et al., 2020; Ahfock and McLachlan, 2023); for example, weed distribution and density estimation (Liu et al., 2024). When obtaining tagged or labeled data is expensive, this can dramatically enhance performance. Another component of machine learning known as reinforcement learning (RL) teaches an agent how to behave and react in a given environment by having it carry out specific tasks and then watching the rewards or outcomes. This technique is already employed in different agricultural domains, such as crop yield prediction (Elavarasan and Vincent, 2020; Iniyan et al., 2023) and a completely autonomous precision agricultural aerial scouting technique (Zhang et al., 2020; Elango et al., 2024).

### 3.3 Concept of classification, clustering, and regression problems

In machine learning, a task is referred to as a classification challenge when it requires allocating data points to a collection of discrete classes such as varieties emitting high or low methane, and a classifier is any algorithm that carries out this kind of classification (Sen et al., 2020), such as cassava disease detection and classification (Bian and Priyadarshi, 2024). Contrary to classification, regression models produce a collection of values that are continuous (Pardoe, 2020; Panigrahi et al., 2023), such as the prediction of yield before the harvest of very early potato cultivars by using a regression model (Piekutowska et al., 2021). Regression problems can frequently be reformulated as classification problems since continuous values can be discretized or thresholded (Greener et al., 2022). Typically based on some metric of data point similarity, in a target dataset, clustering algorithms are applied to predict and group similar data points (Ghosal et al., 2020). These techniques are unsupervised and do not necessitate labeling the instances inside a dataset. For example, according to images of soybean, clustering could predict seed weight (Duc et al., 2023).

### 3.4 Concept of classes and labels

When a classifier returns a discrete collection (set) of mutually exclusive values, such values are referred to as classes. These values are called labels when they do not have to be mutually exclusive. Typically, an encoding is used to represent classes and labels. One essential step in preparing data for machine-learning tasks is encoding categorical variables. It is essential to convert categorical data into a numerical format to make them compatible with machine-learning algorithms. Categorical data are not numerical values, such as categories or text. For example, a place variable with the values first, second and third or a color variable with the values; red, green, and blue is categorical data, which every value denotes a distinct category. There might be an inherent ordering or link between some categories. There is a natural ordering of values for the aforementioned place variable. There is a natural ordering of values for the aforementioned place variable. Due to the fact that the values can be ranked or ordered, this kind of categorical variable is known as an ordinal variable. There are several popular category encoding methods, each combining benefits and drawbacks such as label encoding, ordinal encoding, and one-hot encoding methods. One-hot encoding is one of these techniques, which is most frequently employed (Yu et al., 2022b). When there is no innate link or order among the categories, this encoding performs well with nominal categorical variables (Rodríguez et al., 2018). The distinctiveness of every category is maintained by one-hot encoding. It guarantees that no ordinal link between the categories is assumed by the method. Also, one-hot encoding eliminates the possibility of unintentionally adding biases based on the sequence of categories because each category is represented independently. But when working with categorical variables that have a large number of distinct categories, one-hot encoding can dramatically increase the dataset’s dimensionality. This may result in the curse of dimensionality and have an adverse effect on the performance of the model. Ordinal encoding is used when the categorical feature is ordinal. Every distinct category value in ordinal encoding is given an integer value. For example, in the color categorical data, red is 1, green is 2, and blue is 3. Maintaining the order is crucial in this method and encoding should so take the sequence into account. Equal intervals between categories are assumed by ordinal encoding, yet this may not always be the case in real-world situations (Dahouda and Joe, 2021). Unlike one-hot encoding, ordinal encoding does not increase the dimensionality of the dataset and It saves space and processing time by substituting integers for categorical variables. Label encoding assigns a unique integer value to each category in a categorical feature. This is an easy-to-use strategy that can be helpful when the categories’ order matters. However, because of the allocated integer values, it could create unintentional linkages between categories. For instance, label encoding could assign the values 0, 1, and 2 correspondingly if a categorical feature is small, medium, and large. This would suggest that “large” is twice as significant as “small”, which is probably incorrect. Important point is that the type of categorical variable and the issue those researchers are trying to solve will determine which encoding techniques should be used.

### 3.5 Concept of cost or loss functions

Machine-learning models never produce perfect results; they always deviate from the ground truth or the real world (Ho and Wookey, 2019). Cost or loss functions are the mathematical functions that compute this deviation or, more broadly, the degree of disagreement between the actual and ideal outputs (Uma et al., 2021). Mean squared error loss for regression problems is one example, and, for classification-related problems, binary cross entropy (Nar et al., 2019). A mean squared error loss function calculates the average squared difference between the anticipated value and ground truth. Binary cross entropy is a binary classification problem that must divide observations into one of two labels according to specific criteria [such as healthy leaf and infected leaf (Sarkar et al., 2023)].

### 3.6 Concept of parameters and hyperparameters

In essence, models are mathematical functions that take a collection of imported features and return one or several features or values as an output. Models include adaptable and flexible parameters that can be adjusted throughout the training process to optimize the models’ performance, allowing them to learn from training data (Yu and Zhu, 2020). In a simple regression model, for instance, each feature has a particular parameter that is being multiplied by the value of the feature; these are then integrated and combined to provide a forecast. Hyperparameters are tunable values that are not changed during training and are thus not regarded as a model component. But this nonetheless affects the performance and training model. The learning rate, which regulates the pace at which the model’s parameters are changed during training, is a standard description of a hyperparameter. To simplify it, hyperparameters control a structure and training procedure of machine-learning models, and they might be the number of clusters in K-means clustering, the learning rate in a neural network, or the depth in a decision tree. Hyperparameters, in contrast to model parameters, need to be predefined and cannot be learned during training. A model’s ability to perform well or poorly can be determined by selecting the appropriate collection of hyperparameters. Therefore, choosing the set of hyperparameters that result in the best possible model performance is known as hyperparameter tuning. Depending on the type of model being trained, different sorts of hyperparameters may be employed including learning rate, number of epochs, batch size, number of hidden layers and units, regularization parameters, momentum, and activation function. Several tools are developed for model tuning and hyperparameter optimization such as Ray Tune (Shin et al., 2020), Optuna (Akiba et al., 2019), HyperOpt (Bergstra et al., 2015), and AWS Sage Maker (Das et al., 2020).

### 3.7 Splitting target data into training, validation, and testing sets

**Figure 2:**
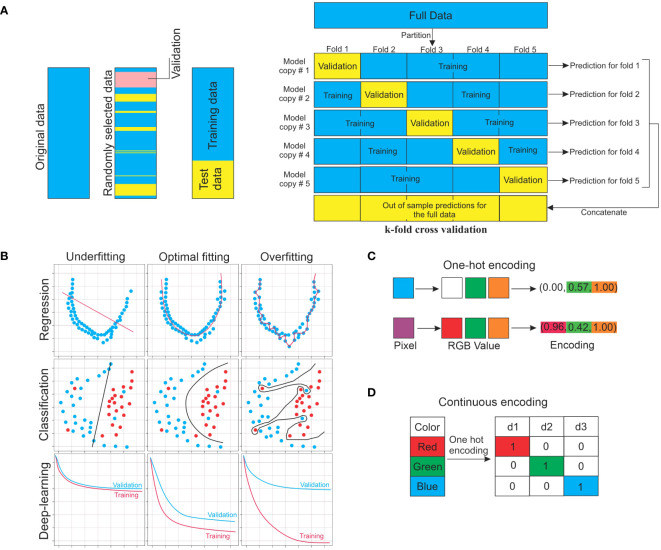
ML approaches for training. **(A)** Target data for machine learning should be split into training, validation, and testing sets. The training set is used to train the model directly. With the validation set, the training set is monitored. Test data are used to assess the performance of the model. The *k*-fold cross-validation approach is also used for validation. **(B)** Concept of underfitting and overfitting. **(C)** One-hot encoding is a process by which categorical variables are converted into a form that could be provided to ML algorithms to do a better job in prediction. **(D)** Numerical data can be represented in a way that machine-learning algorithms can understand using continuous encoding. In the given example, the RGB (R: red, G: green, and B: blue) are shown as the specific values of pixels in the targeted images.

Models need to be trained, which is the process of automatically modifying model parameters to enhance performance before they can be used to generate predictions (Mathai et al., 2020) This means altering the parameters in a supervised learning setting to minimize the average value of the loss or cost function and improves model performance with a training dataset. Typically, a separate validation dataset tracks but does not alter the training process to detect any overfitting (Twomey and Smith, 1997). Even if a cost function does not run on ground truth outputs in unsupervised scenarios, it is nonetheless decreased. After training, the model can be evaluated using data not used during training (**Figure 2A**) (Eelbode et al., 2021). For a general overview of the training procedure and instructions on how to divide the target dataset into training set and testing set. **Figure 2** illustrates the principal notions for the training of models and displays a flowchart to aid in the whole procedure.

### 3.8 Concept of overfitting and underfitting

For a model to be predictive of unobserved (non-training) data, it must be fitted to training data to grasp the entire connection among all possible variables inside the dataset. The common reasons that a machine-learning model performs poorly are challenges, two key concepts in the field of machine learning (**Figure 2B**). An overfitted model (often caused by having too many parameters) can generate outstanding output on trained data but will produce adverse outcomes on unobserved data. High variance and low bias might lead to overfitting. The training dataset will have zero prediction error since the overfitted model goes through each training point perfectly, as shown in **Figure 2B**. Conversely, an underfitted model cannot accurately represent the connections between the data variables. This can result from an improper model type selection, inaccurate or inadequate data assumptions, a high bias, or a low variance procedure (**Figure 2B**).

### 3.9 Concept of the bias-variance tradeoff

The inductive bias of the model is the collection of assumptions made by the learning algorithm that leads it to prefer one solution to a learning problem over another (Baxter, 2000). This could be understood as the model favoring one learning solution over another. This choice is frequently encoded into the model by using a specific loss function and/or its particular mathematical form. Various model types have distinct inductive biases that make them more appropriate and they often perform better for specific categories of data. The tradeoff between variance and bias is another crucial concept in machine learning. The general argument is that a model with a large bias places more restrictions on the trained model. Conversely, the low-bias model decreases the number of assumptions about the model property, and it is theoretically capable of modeling a large range of function kinds (Neal, 2019). The amount that the trained model varies when it is trained on various training datasets is indicated by the variance of the model. Ideally, models should have low variance and bias, but these goals frequently conflict with each other, given that a model with low bias will learn distinct signals on separate training sets. To prevent either overfitting or underfitting, it is essential to manage the bias-variance tradeoff.

## 4 Overview of ML procedures and required concepts

**Figure 3:**
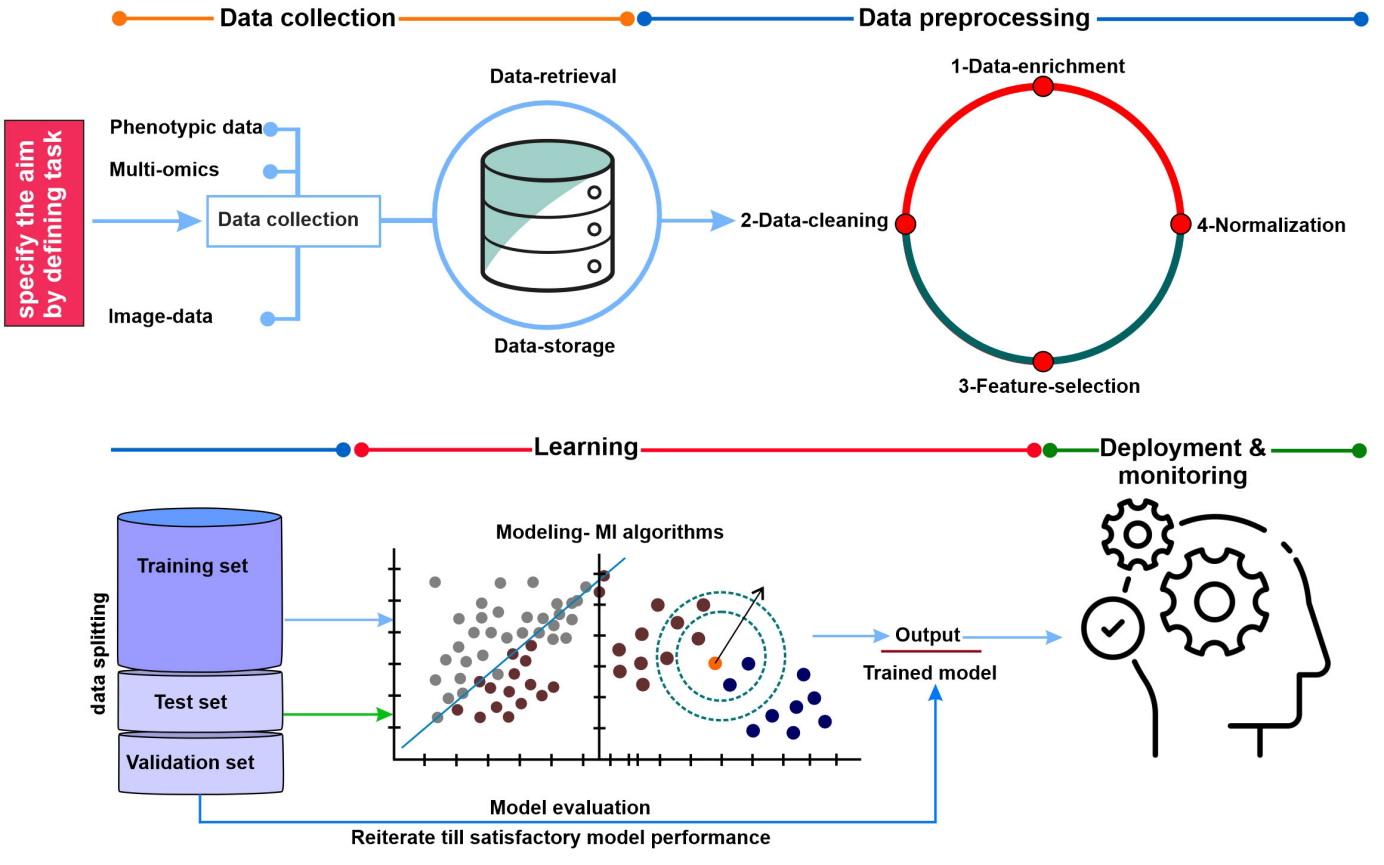
The graphic illustrates the general process for data collection, training, testing, and validation of machine-learning and model evaluation methods. Data collection: collecting data from various data sources related to crop improvement and development. Data processing: the most important step in the ML space is data cleaning and pre-processing. Before beginning the analysis of the best algorithm for the provided data set, we must comprehend the data set and select the cleaning or pre-processing procedures to obtain the best possible outcomes. Learning (Model building and selection of ML algorithm): This is a crucial stage that will soon bring the process to an end. Thus, we must choose our models carefully because we will select one as the ultimate model to solve the provided business challenge. Model evaluation: Model evaluation validates a target model with some standardized mathematical formulae or methodology. Deployment and monitoring: the selected ML model is authorized for deployment in the production environment after its performance satisfies the requirements, and the performance and behavior of the selected model in the real world are continuously observed, analyzed, and assessed through the process known as model monitoring.

This section is a concise survey of the procedures that should be followed for training an ML model (**Figure 3**). Surprisingly, little advice is given for the selection of specific models and methods of training (Bengio, 2012; Greener et al., 2022). The first step is to understand the problem, the nature of the imported data, and the final goal of the prediction, which should come before writing any ML algorithms. This step is essential to have a comprehensive understanding of the crop improvement aspect of the problem or question: for example, knowing the sources of noise and the origin of the target data. Understanding the computational storage of the inputs and outputs is also crucial. The following questions could be addressed: Are they adjusted (normalized) to avoid an excessively high effect of one attribute on prediction? Do they have continuous or binary encodings? Are some entries repeated? Are some data pieces missing (NaN)?

In the following step, the collected data (target dataset) must be divided into the first training dataset, the second for validation, and finally the testing dataset (**Figure 2A**). The training dataset is used for training an ML algorithm. In contrast, the test dataset (holdout set) is used to evaluate the resulting model (to estimate how well the model performs on unseen data). This idea is further used in the model selection part of the training procedure, which might lead to allocating a part of the training dataset as a validation set while the rest of the training data are used for training proper. Using the training set, the parameters of the specific model are updated during the training procedure. Usually, 10% of the available data are split and considered validation data to oversee instruction (training) performance, thus avoiding overfitting of the target model, and select hyperparameters (previously explained) based on datasets for training. Frequently, *k*-fold cross-validation is used in this step. Typically, 10% to 20% of the total dataset is dedicated as a test dataset to evaluate the expected real-world performance of the target model by assessing how well it performs on data that were not used for training or validation. To prevent adjusting the model to match the test set, there should be only one-time use of the test set in the later stages, if possible (Hastie et al., 2009; Bzdok et al., 2018). Selecting a model comes next, depending on the dataset type (nature of data) and the kind of anticipation being formed. This is conceptualized and made concise in **Figure 3**. To raise the overall accuracy of the undertaken model, the ensemble model averages the outputs of several comparable models that could be considered. Finally, evaluating the model’s accuracy in the dedicated test dataset is crucial.

## 5 Conventional machine learning

**Figure 4:**
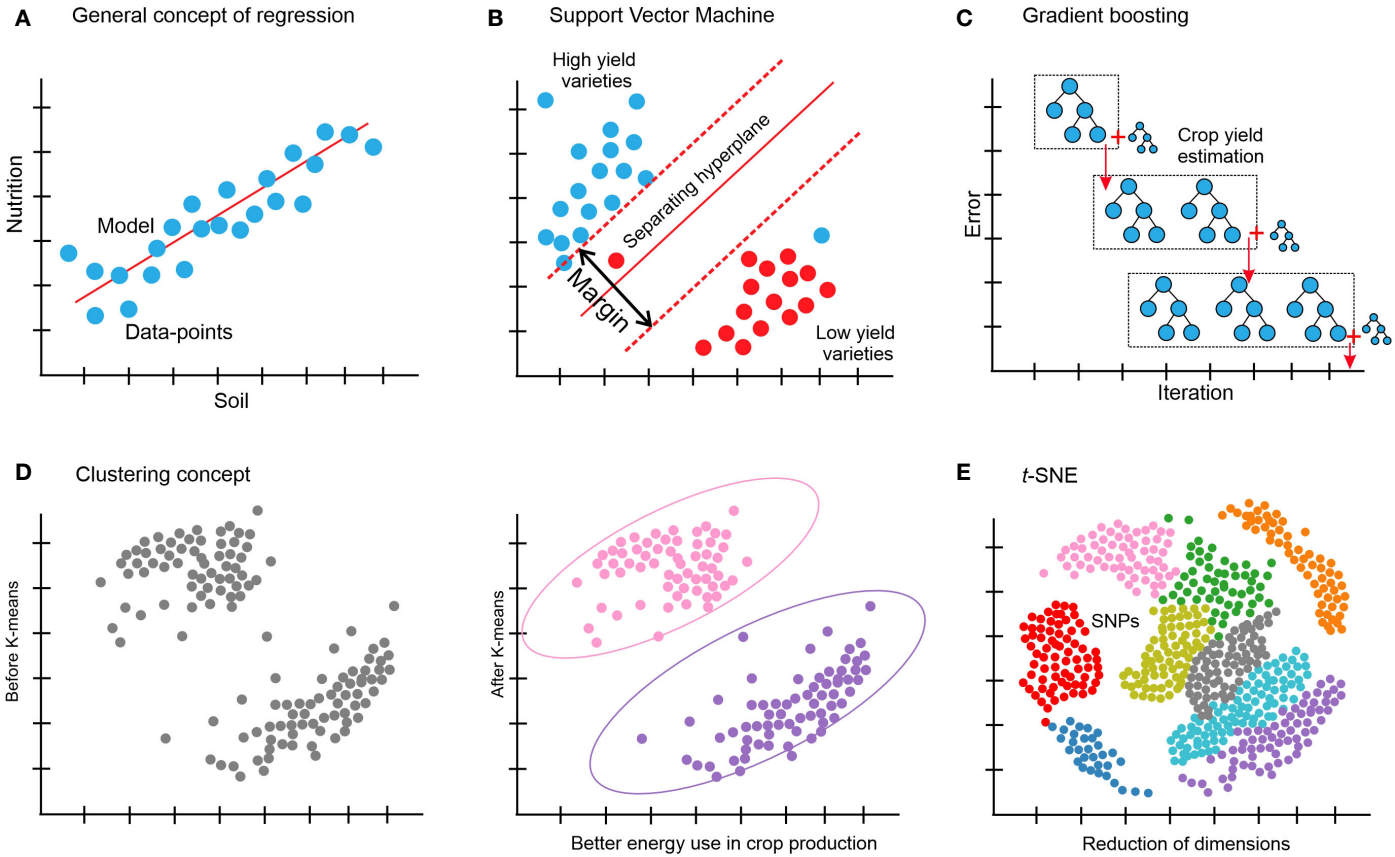
**(A)** Regression is the link between a single and/or several independent variables, also known as features, and a dependent variable (the observable attribute) is determined by using regression. A straightforward example is the prediction of crop yield based on one or some of the phenotypic features. **(B)** SVM: a support vector machine divides the original input data into several categories by creating a gap as large as feasible between the data in each converted version. One example is a prediction of whether a variety of a specific crop is a low- or high-yield variety. **(C)** Gradient boosting makes predictions by combining several weak prediction models, most often decision trees; for example, the prediction of sugarcane yield grade. **(D)** Clustering: using one of several algorithms, based on related objects; for example, better energy use in crop production. **(E)** t-distributed stochastic neighbor embedding (t-SNE), for example, dimensionality reduction of crop genotypic (SNP) data.

This section investigates several essential and traditional machine-learning techniques, focusing on their advantages and disadvantages. **Table 1** presents a comparison of several machine-learning techniques along with some applications for crop improvement and production. **Figure 4** illustrates a few of the conventional machine-learning techniques. To train these models, several software programs have been available, such as Caret in R (Kuhn, 2008; Dege and Brüggemann, 2023), MLJ in Julia (Blaom et al., 2020), and scikit-learn in Python (Pedregosa et al., 2011; Rajamani and Iyer, 2023). When developing machine-learning algorithms for crop improvement-related data, conventional machine learning is typically the first area to investigate to find the most appropriate solution for a given problem. Deep learning is currently prevalent and has the potential to be a robust and valuable method. It is still restricted to the application domains where it performs well, though, such as when a vast quantity of data are accessible, such as extreme data points, when there are several features on each data point or when the features have a lot of structure (Greener et al., 2022). Drone images from crop fields (Killeen et al., 2024; Sahoo et al., 2024) and genotypic data (SNPs) (Uppu et al., 2016) are two examples of agricultural data for which deep learning could be effectively used. Even when the other two conditions are satisfied, deep learning may not be the best option because of the need for vast volumes of data. Technically, conventional approaches build and evaluate solutions for a particular problem far more quickly than deep learning. When compared to more conventional models such as random forests and support vector machines (SVMs) (Hastie et al., 2009), creating the architecture and training a deep neural network might be a computation-intensive and costly process (Sejnowski, 2018). For a given agricultural prediction problem, even if deep learning seems theoretically doable, it is usually wise to train a conventional technique and evaluate it against a model based on neural networks such as ANN (artificial neural network), if at all possible (Smith et al., 2020). Conventional approaches usually assume that every sample in the collection has the same number of characteristics, which is not always feasible. Using SNP data with varying lengths for each case is a clear illustration of this problem. The data can be adjusted using basic techniques such as windowing and padding to make them all the same size and employing standard ways with them. Padding refers to the process that can add zero value to each example up to making the size of each of them equal to the most prominent example in the target dataset. Conversely, the windowing approach condenses each sample to a specific size (Chrysostomou et al., 2011).

**Table 1:** Comparison of different machine-learning methods.

Method	Data types	Advantage	Disadvantage	Agricultural example
Support vector machine (SVM)	- Supervised learning (labeled) - Definite number of features	Capable of doing regression and classification both linearly and non-linearly	Large dataset scaling is frequently challenging	- Land suitability - Crop yield prediction (Lingwal et al., 2024) - Classification of weeds and crops based on digital images (Ahmed et al., 2012; Agarwal, 2024)
Ridge regression	- Supervised learning (labeled) - Definite number of features	- Prevent overfitting - Simple to train - A decent reference point (benchmark)	- Unable to understand the sophisticated relationship between features - Having overfits with an excessive number of features	- Genotype-specific grain yields of wheat (Herrera et al., 2018) - Predicting soil nutrition (Sudha et al., 2022)
LASSO regression	- Supervised learning (labeled) - Definite number of features	-Prevent overfitting -Remove highly inter-correlated features in data	- Chooses just one feature from a set of related features - Certain features might have significant bias	- Forecasting crop yield (Kashyap et al., 2024) - Wheat yield prediction (Shafiee et al., 2021)
Random forest	- Supervised learning (labeled) - Definite number of features	- Effective with big datasets - Discover how crucial each feature is to the forecast - More accessible for training and adjusting since it is less susceptible to feature normalization and scaling	- Not as suitable for regression - Interpreting several decision trees might be challenging.	- Crop yield predictions (Jeong et al., 2016; Basha et al., 2020; Dhillon et al., 2023)
Gradient boosting (such as XGBoost)	- Supervised learning (labeled) - Definite number of features	- Discover how crucial each feature is to the forecast - Easier to train and adjust since it is less susceptible to feature normalization and scaling	- Not as suitable for regression - Might find it difficult to learn information when there is noise	- Yield estimation (Huber et al., 2022) - Maize variable-rate seeding decision (Du et al., 2022)
Clustering	- Unsupervised learning (unlabeled) - Definite number of features	- Performance could be evaluated using accessible cluster validation metrics - Good clustering for low-dimensional data is readily observable	- Results from noisy datasets could occasionally be contradicting - Certain techniques have trouble scaling to huge datasets	- Crop yield predictions (Vani and Rathi, 2023) - Better energy use in crop production (Khoshnevisan et al., 2015; Wu et al., 2024)
Reduction of dimensions	- Unsupervised learning (unlabeled) - Definite number of features	- Gives clear ideas through visualization of datasets - Evaluations of goodness-of-fit are often provided to evaluate performance	- For specific techniques, scaling to vast numbers of samples is challenging - Preserving both local and global data differences is challenging	Dimensional reduction from genotypic data (SNPs) (Heffner et al., 2009; Evamoni et al., 2023)
Multi-layer perceptron	- Supervised learning (labeled) - Definite number of features	- Applies to intricate non-linear issues - Performs well with considerable data input - Quickly makes predictions following training - Even with fewer data, the same accuracy ratio can be attained	- The degree to which the dependent variable impacts each independent variable is unknown - Completing computation takes a lot of effort and time - Training data quality is critical to the correct operation of the model	- Predicting maize yield (Ahmed, 2023) - Predicting soil electrical conductivity (Mosavi et al., 2021)
Convolutional neural network (CNN)	- Grid-based spatial data arrangement	- High precision - Specifically made to handle image datasets - Able to derive spatial characteristics from a hierarchical matter	- Hefty computational expenses - Needs a huge dataset - Huge parameter size makes it challenging to optimize	- Crop classification (Mazzia et al., 2019; Kavitha et al., 2024) - Crop yield prediction (Nevavuori et al., 2019; Kolipaka and Namburu, 2024)
Recurrent neural network (RNN)	Data in sequential format (genotype data or time series)	- Capable of handling input of any length - For lengthier input, the model size would not increase - Sequence data format is seen in many agricultural domains	- Recurrent processing is time-consuming - High memory needs for computing	- Crop improvement (Gopi and Karthikeyan, 2024) - Crop yield prediction (Gopi and Karthikeyan, 2024)
Graph convolutional network	Connections and relationships between entities define the data	- Observes graph connection to identify patterns, allowing the predictor to use the most pertinent links	- More complex designs are challenging to train - High memory needs for computing	- Weed and crop recognition (Jiang et al., 2020; Pandey et al., 2024) - Crop recommendation systems (Ayesha Barvin and Sampradeepraj, 2023)
Autoencoders	Supervised and unsupervised data (labeled and unlabeled data format)	- Noise identification ability - Effective in extracting features	- Restricted ability - The challenge of interpreting the outcome - Other datasets might not benefit from using latent space unique to the training set’s data - Uses more memory resource	- Plant disease detection (Boukhris et al., 2024) -Crop classification (Guo et al., 2020; Cui et al., 2023)

### 5.1 Application of regression and classification models

Regarding regression problems such as those depicted in **Figure 4A**, ridge regression (a type of linear regression) is frequently a valuable place to start when building and developing a model since it could offer a quick and clear baseline for a particular responsibility. The value of one variable can be predicted by using linear regression analysis according to the value of another variable (Su et al., 2012). On the other hand, when a model relies on as few features as possible from the given data, then other variations of linear regression, such as elastic net regression (Zou and Hastie, 2005) and LASSO regression (Tibshirani, 1996), are also worthy of consideration. Since the correlations between the characteristics in the data are frequently non-linear, using a model such as an SVM is usually a better option in these situations (Noble, 2006), as shown in **Figure 4B**. SVMs are a practical kind of classification and regression model that convert non-separable problems into easier-to-solve separable problems by using kernel functions. A kernel function is a technique for transforming input data into the format needed for data processing. Both non-linear (a statistical method called non-linear regression is used to model non-linear relationships between independent and dependent variables) and linear regression could be carried out with SVMs based on the kernel function that was applied (Ben-Hur et al., 2008; Ben-Hur and Weston, 2010; Kircher et al., 2014). To quantify, the best idea is to train an SVM through a kernel of a radial basis function and a linear SVM can be used from a non-linear model, if any. Numerous models that are often employed in regression could be used in classification as well. Another acceptable default starting point for a classification problem is to train an SVM based on the kernel function and a linear SVM. *k*-nearest neighbors classification (also known as k-NN or KNN) is a further technique that could be used (Bzdok et al., 2018). A non-parametric supervised-learning classifier, the *k*-nearest neighbors method employs closeness to classify or anticipate how a single data point will be grouped (Peterson, 2009). XGBoost (**Figure 4C**) (Chen and Guestrin, 2016; Olson et al., 2018) and random forests (Wang and Zhang, 2017) are examples of ensemble-based models, which provide another family of resilient non-linear techniques. These techniques are effective non-linear models offering feature significance estimations and frequently just need minor adjustments to the hyperparameters. There are often an overwhelming number of variations among the several models available for regression and classification. It can be misleading to try to forecast how well-suited a specific method will be to a given issue in advance; instead, it is usually wiser to use an empirical approach to identify the optimum model via trial-and-error methods. Swapping out these model versions often involves only one line of code change thanks to a novel and robust machine-learning library such as scikit-learn (Pedregosa et al., 2011), which can efficiently run in a Python environment. To find the best approach overall, it is an excellent strategy to optimize and train several of the previously described techniques, and then compare the results on a different test set to see which method performed the best on the validation set.

### 5.2 Application of clustering models

Like many other clustering algorithms (**Figure 4D**), k-means is a powerful multi-purpose clustering technique that requires the number of clusters to be specified as a hyperparameter (Jain, 2010). An alternate method that is not necessary for a predetermined number of clusters is DBSCAN (Ester et al., 1996). For datasets with plenty of features, dimensionality reduction can also be done prior to clustering to enhance performance.

### 5.3 Dimensionality reduction

High-dimensional data can be transformed into a lower-dimensional format while preserving the different connections and interactions between the data points and pieces using dimensionality reduction techniques. Although more dimensions could be used in machine learning, two or three dimensions are often selected to enable data visualization on several axes. These methods include data transformations that are both linear and non-linear. Principal component analysis (PCA) (Jolliffe and Cadima, 2016) and *t*-distributed stochastic neighbor embedding (*t*-SNE) (Van der Maaten and Hinton, 2008) are some of the examples common in the agriculture domain for dimensionality reduction. The circumstance determines which technique to apply. PCA is based on a linear combination of input features; each component preserves the global connections between the data points and could be explainable, implying that it is simple to identify the characteristics that contribute to data diversity. *t*-SNE is a versatile technique that can uncover structure in complicated datasets and more robustly maintain local links between data points (**Figure 4E**).

## 6 Concept of artificial neural networks

**Figure 5:**
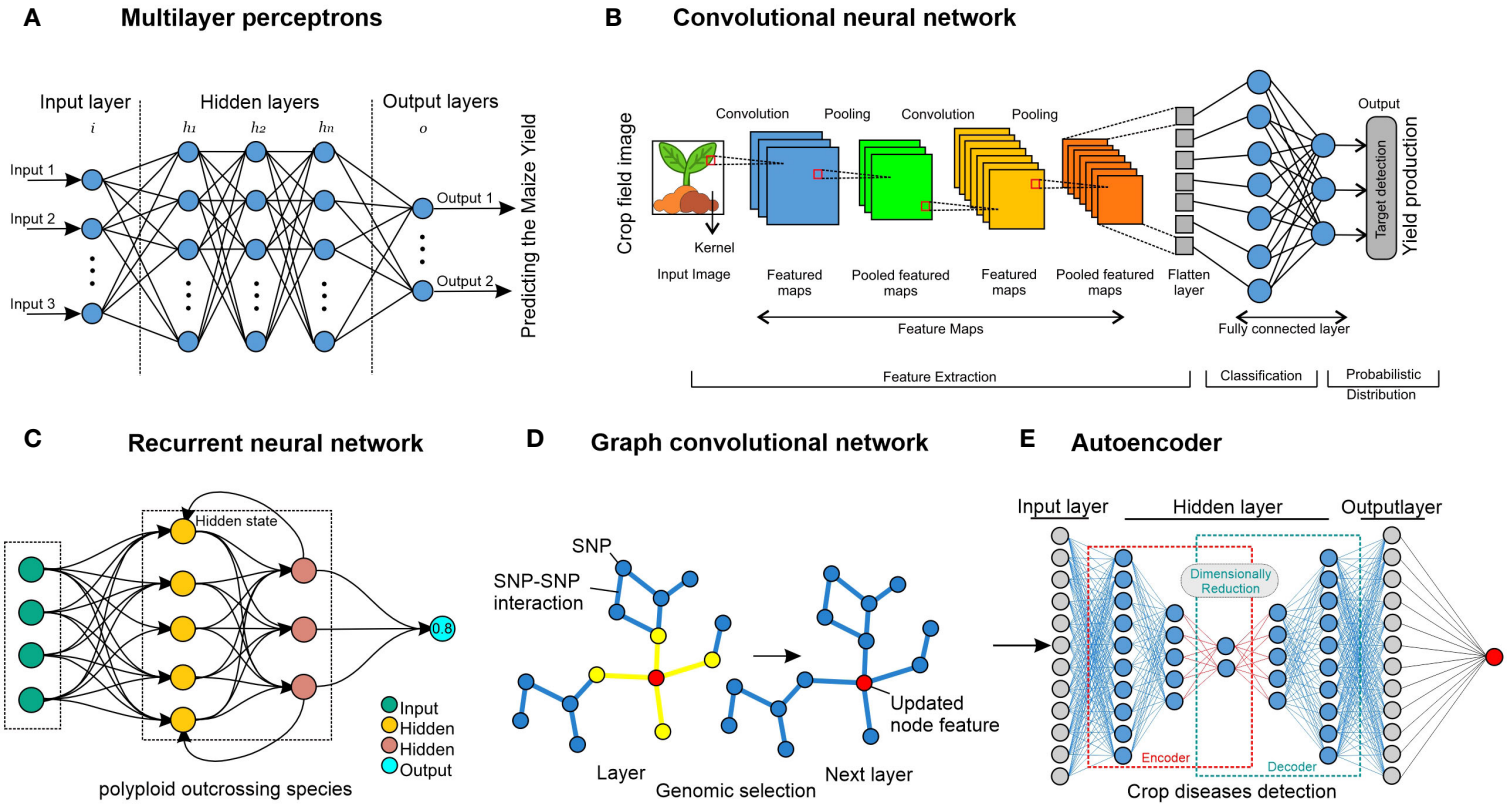
Neural network approaches. **(A)** MLP comprise nodes, which are represented by circles and can be either internal (hidden) values or output values. Layers of nodes are created by connecting each node in one layer to every node of other layers, signifying that the links represent learned parameters. For instance, the maize crop has been used for predicting yield (Ahmed, 2023). **(B)** To compute the values in the subsequent layer, a CNN employs filters that traverse the input layer. Since the filters work throughout the layer, parameters are shared, making it possible to identify related things wherever they may be. Although 1D and 3D CNNs are also used in crop improvement, 2D CNNs demonstrated operating on images of crops. 1D and 3D CNNs have been used for crop and crop-land classification (Ji et al., 2018; Liao et al., 2020; Liu et al., 2023). **(C)** An RNN is a deep-learning model trained to interpret and translate a given set of sequential data inputs into a predetermined set of sequential data outputs. It is used for weed detection for crop improvement (Brahim et al., 2021). **(D)** A GNN uses data from linked nodes of graph format data. It can be used for genomic selection in crops, and it has been used for predicting crop variety yield (Yang et al., 2023). **(E)** An autoencoder is composed of an encoder neural network that transforms an input into a latent representation with lower dimensions, and this hidden representation is transformed back into the original input by using a decoder neural network. This method has been used in crop disease detection for crop improvement (Abinaya and Devi, 2022).

The mathematical principle of artificial neural networks (ANN) has been conceptualized by following and understanding the behaviors and connectivity of human neurons in the human brain. It was created initially to study the workings of the brain (Crick, 1989). The significant advances in deep neural network training and architecture over the past few decades have increased interest in neural network models (LeCun et al., 2015). The following section covers the fundamentals of neural networks and common varieties used in research on crop improvement. **Figure 5** displays some of these concepts.

### 6.1 Concept of neural network fundamentals

The capacity of neural networks to approximate functions universally is one of their primary characteristics; this implies that, with minimal presumptions, any mathematical function can be accurately approximated to any degree by a neural network that is set up appropriately. The fundamental units of every neural network model are artificial neurons. A mathematical function that translates (converts) inputs to outputs in a certain way constitutes an artificial neuron (Wu and Feng, 2018). Any number of input values can be fed into a single artificial neuron, which then uses a predetermined mathematical function to produce an output value. Artificial neurons are layered and the output of one layer is the input of the next, which forms a network. In the following subsections, we present several methods for configuring artificial neurons, sometimes called neural network architectures. Combining several architectural styles is also popular. For instance, fully linked layers are typically used to provide the final classification output in a CNN (convolutional neural network) used for classification.

### 6.2 Concept of multi-layer perceptrons

A feed-forward ANN (artificial neural network) having several layers, comprising an input layer, one or several hidden layers, and an output layer, is called a multi-layer perceptron (MLP) (**Figure 5A**). Every layer is wholly interconnected with every other layer. The term “perceptron” was initially established by Frank Rosenblatt (Seising, 2018). The fundamental building block of an artificial neural network, the perceptron, specifies the artificial neuron inside the network. Activation functions, node values, inputs, and weights are all used in this supervised learning technique to determine the output. The forward direction is supported by the MLP neural network. Every node has complete network connectivity. Only in the forward direction does each node transmit its value to the next node. Back-propagation is a method used by the MLP neural network to back propagate the error in order to optimize the weights and unit values.

### 6.3 Concept of convolutional neural networks

CNNs are developed mainly to use image data format, and the fundamental component of a CNN is the convolutional layer (**Figure 5B**). Three things are needed: a feature map, a filter, and input data (Li et al., 2021). Suppose the input will consist of a color picture, a 3D matrix of pixels. As a result, the input will have three dimensions: height, width, and depth, which match the RGB color space of a picture. CNNs are equipped with a feature detector, which could also be called a kernel or filter. This detector traverses the receptive fields of the image and determines (Bouvrie, 2006). A convolution is the name given to this procedure. CNNs can be set up (configured) to function well with various spatially structured datasets. A 1D CNN, for instance, would contain filters that move in only one way. Data with one spatial dimension would be a perfect fit for this kind of CNN (Tang et al., 2020), such as genotypic (SNP) data from rice varieties. Digital images are examples of data with two spatial dimensions that 2D CNNs can process (Hara et al., 2018). Volumetric data, such as multi-temporal remote-sensing images, are what 3D CNNs use to function (Ji et al., 2018). Significant progress has been made in crop improvement for various datasets using CNNs (Jiang and Li, 2020). Crop classification (Durrani et al., 2023), crop yield prediction (Nejad et al., 2022), and maize seedling recognition (Diao et al., 2022; Wei et al., 2024) are some examples of CNN models for crop improvement, and they now frequently surpass skilled human performance.

### 6.4 Concept of recurrent neural networks

RNNs are the most suitable approach with data organized into sequences, where each point in the series has some semblance of dependence or connection with the previous one (at least conceptually) (Greener et al., 2022), as seen in **Figure 5C**. The primary use of this approach is probably in NLP (natural language processing), which considers text a succession of characters (Medsker and Jain, 2001). One kind of RNN that can retain the outputs of each node for extended periods is called long short-term memory (LSTM) (Goodfellow et al., 2016). In other words, RNNs are modified to build LSTM networks, which provide better recall of previously learned data. Using back-propagation, they train the target model. When dealing with time delays of undetermined length, LSTM is a robust tool for classifying, processing, and predicting time series. Thus, once data are presented in an orderly structure, such as time sentences, LSTM can frequently be employed in different fields such as NLP and time-series analysis (Abdel-Nasser and Mahmoud, 2019). In the crop domain, RNNs are used extensively for crop improvement, such as land cover classification (Sun et al., 2019), prediction of crop biomass (Masjedi et al., 2019), and land cover and crop classification (Mazzia et al., 2019; Abidi et al., 2023; Moharram and Sundaram, 2023).

### 6.5 Principle of graph neural networks

Graph neural networks (GNNs) are especially well-suited for data that lack a clear apparent structure, such as a picture. Still, they are made up of things connected by randomly determined interactions or relationships (Battaglia et al., 2018). Such applications relevant to crop improvement include weed and crop recognition in smart farming (Jiang et al., 2020; Pandey et al., 2024) and crop recommendation systems (Ayesha Barvin and Sampradeepraj, 2023; Ge et al., 2024). In computer language, a graph is merely a representation of this kind of data, and every graph has a collection of nodes or vertices and a collection of edges that show different types of relationships or connections between the nodes. As seen in **Figure 5D**, when each feature of the nodes is updated across the network, neighboring nodes are considered. The node features in the final layer are then used as the output or merged to generate an output for the entire graph. Graphs illustrating various correlations could use data from several sources to make predictions. Graph Nets (Gao and Ji, 2019) and PyTorch Geometric (Fey and Lenssen, 2019) are some of the most popular programs used to train GNNs.

### 6.6 Autoencoder networks

Autoencoder is used for unsupervised learning or the efficient coding of unlabeled input (Bank et al., 2023). The autoencoder method can learn two tasks: transforming input data by using an encoding function and recreating the input data from the encoded representation by a decoding function (**Figure 5E**). An alternative perspective is that the encoder attempts to compress the input and the decoder attempts to decompress it. Concurrent training is applied to the encoder, latent representation, and decoder (Doersch, 2016). Predicting the imposing of a structure on the latent space and the degree of similarity between two data points helpful for prediction tasks are two examples of applications. This approach has been used in several domains of crop improvement, such as crop classification (Bhosle and Musande, 2022; Cui et al., 2023) and crop mapping (Hamidi et al., 2021; Madala and Prasad, 2023; Hamidi et al., 2024).

### 6.7 Neural network improving and training

**Figure 6:**
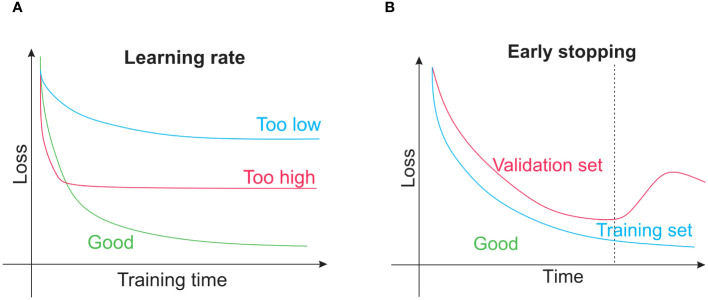
**(A)** The learning rate concept is that, when training a neural network or other conventional techniques such as gradient boosting, the learning rate of the model controls how quickly parameters that are learned are changed. **(B)** Early stopping is a regularization technique that helps prevent overfitting when training learners using gradient descent or other iterative methods.

Several issues are unique to neural networks as they are far more sophisticated than conventional machine-learning techniques. It is frequently a good idea to train a neural network on a single training sample after deciding that it is the best model for the desired application for instance, a single image. The trained model is not helpful in forecasting, whereas it is adequate for exposing programming flaws (errors). As the network retains only the input, the training loss function ought to rapidly approach zero. If not, either the algorithm is not sophisticated enough to represent the input data or there is probably a mistake in the code. The network can begin with training on the whole training set after passing this fundamental debugging test when there is a minimum in the training loss function. It might be necessary to adjust hyperparameters such as the learning rate for this, as shown in **Figure 6A**. Overfitting of the network can be identified by tracking loss on the training dataset and validation dataset, where loss on the training set starts to rise and loss on the validation set keeps becoming less. At that moment, training is often discontinued, a procedure called early stopping, as shown in **Figure 6B**. A neural network overfitting indicates that the model’s capacity to generalize to new data is beginning to wane as it starts to memorize only the features of the training set. Although early stopping is an intelligent strategy for avoiding this, other training approaches could be employed, such as dropout methods or model regularization. Nodes within the network are arbitrarily disregarded to compel the network to discover a more reliable prediction method incorporating more nodes. TensorFlow (Abadi et al., 2016) and PyTorch (Paszke et al., 2019) are well-liked neural network training programs. Neural network training is computationally intensive and often calls for a tensor processing unit or graphics processing unit with enough RAM (random-access memory) because using these devices could accelerate work 10 to 100 times faster than using a regular CPU (central processing unit). This acceleration is necessary for training massive datasets and for the larger models that have demonstrated success in recent years. Nevertheless, using a model that has already been trained is typically much quicker and this could frequently be accomplished with a simple CPU. For researchers without access to a GPU (a graphics processing unit is on-demand computing services) for training, cloud computing options are available from popular suppliers, and thus it is essential to remember that for simple tasks. Python code could be freely tested on graphics or tensor processing units using Colaboratory (Colab). A practical method to get started with deep learning based on Python is to use the Colab environment.

## 7 Challenges of machine learning for crop improvement and production-related data

The enormous diversity of agricultural and crop domain data is one of the significant challenges in modeling, and these data are also generated from different nature domains. Crop improvement-related data can be yield-related data, land-related data, crop-development-related data, crop-disease-related data, or even microorganism data. Most of them can be along with genotypic and transcriptomic data such as SNPs or RNA-seq data and/or high-resolution images, 3D structures, or gene expression profiles over time, and different interactions of networks are some examples of these data formats and natures. A summary of recommended techniques and crucial factors for several crop-improvement data kinds is provided in **Table 2**. Because of the variety of data formats encountered, processing crop-improvement-related data frequently calls for customized solutions. Because of this, it is challenging to provide ready-made solutions or even broad suggestions for applying machine learning in various fields of study. However, for machine learning to be used successfully in crop improvement and agriculture, as well as more broadly, a few common challenges must be considered.

**Table 2:** Suggestive strategies for applying machine-learning techniques to varied datasets related to crop improvement.

Input data format	Recent instances of prediction tasks	Suggested models	Challenges for implementing
Images	- Crop disease monitoring (Bouguettaya et al., 2023; Zhang et al., 2024) - Crop protection (Gauriau et al., 2024) - Yield prediction (Zanella et al., 2024) - Stress detection (Butte et al., 2021; Gholap et al., 2024) - Crop growth (Memon et al., 2021; Attri et al., 2024) - Species detection (Picon et al., 2022) - Water management for crop improvement (Jain et al., 2021; Meenal et al., 2024)	- Autoencoders - 2D CNNs - Conventional techniques based on image features	- Difficult to have reliable dataset - Produces massive amount of data, which are difficult to maintain - Prediction could be affected by systematic variations in data collection - Expensive to provide the dataset - Data collection is an expensive process
Phenotypic data	- Yield prediction (Cao et al., 2021; Dhaliwal and Williams, 2024) - Crop productivity (Mochida et al., 2019) - Species recognition (Chen et al., 2023; Rangarajan et al., 2023) - Crop seed germination (Colmer et al., 2020; Duc et al., 2023)	- SVM - KNN - ANN/SNKs - 1D CNNs - K-means clustering - Conventional machine-learning models - Deep feed-forward multi-layer perceptron	- Lack of access to reliable datasets - Lack of uniform protocol for data collection - High noise
Geographic and climatic data	- Crop production (Alif et al., 2018; Dhillon et al., 2024) - Forecasting crop yield (Veenadhari et al., 2014; Kheir et al., 2024) - Crop yield change projections (Li et al., 2023) - Crop modeling with machine learning (Zhang et al., 2021b; Mousavi et al., 2024) - Crop selection (Yesugade et al., 2018; Kamatchi and Muthukumaravel, 2024) - Crop evapotranspiration (Yamaç and Todorovic, 2020; Du et al., 2024)	- SVM - ANN - 1D CNNs - LSTM RNN	- Different performance of the trained model in unknown regions - High noise
Genotypic data	- Crop improvement (Tong and Nikoloski, 2021; Guo and Li, 2023) - Identifying true single nucleotide polymorphisms (Korani et al., 2019; Sehrawat et al., 2023) - Phenotype prediction (Danilevicz et al., 2022) - Uncovering QTL (Yoosefzadeh-Najafabadi et al., 2022) - Introducing new candidate genes for specific traits (Mora-Poblete et al., 2023)	- Autoencoders - 1D CNNs - SVM - ANN - CNN - GNN - Graph embedding	- Because datasets are dispersed and stored in different places, they are difficult to obtain - Data leaks might make validation challenging

### 7.1 Availability of high-quality data

Since data quality directly affects the functionality, precision, and dependability of ML models, it is essential to the field of artificial intelligence. Models that use high-quality data are more predictive and yield more consistent results. There are some main challenges for insuring data quality in ML including Data collection; the problem facing crop research institutes is obtaining high-quality data from a variety of sources. Ensuring that every data point followed to the same criteria for collecting data and getting rid of redundant or contradicting data is difficult. Data labeling; for training purposes, machine learning algorithms require labeled data; yet, manual labeling is error-prone and time-consuming. Accurate labels that accurately represent real-world conditions are the difficult part. There are some tools and software to generate ground truth data for ML specifically for certain domain such as ROOSTER (image labeler and classifier) (Tang et al., 2023), Bounding boxes (Osman et al., 2021), Polygons (Li et al., 2012), and Polylines (Opach and Rød, 2018). Data security and storage; preserving the integrity of data also entails shielding it from potential corruption and unwanted access. Also, it is essential for agricultural research institutions to have reliable and secure data storage. Data governance; it is difficult for many research facilities to put in place data governance structures that adequately handle problems with data quality. Errors, inconsistent data, and segregated data can result from improper data governance. Also, there is a need for more open-access datasets and standardized data collection protocols to facilitate ML research It is essential to develop more reliable and accurate data collection methods to ensure high-quality data for ML research for crop development improvement.

### 7.2 Accessibility of data

Compared to other domains, agricultural and crop-related data have little publicly available data. The selection of strategies that could be applied successfully is significantly influenced by the amount of data available for a particular import data format. Technically, researchers are effectively compelled to employ more conventional machine-learning techniques when limited quantities of data are available because the accuracy of these approaches is more reliable in these particular cases. Deep neural networks and other highly specified models can be explored once more significant quantities of data are available. For supervised machine-learning approaches, it is essential to take into account the relative quantities of every ground truth label included in the dataset. If some labels are insufficient, more data will be needed for machine learning to function (Wei and Dunbrack, 2013; Alzubaidi et al., 2023).

### 7.3 Model interpretability

Researchers often aim to determine why a particular model predicts some subjects in a certain way and why this particular model works in certain situations and is not accurate in other conditions. Putting it in another way, rather than focusing just on correct modeling, agri-researchers are typically interested in identifying the mechanisms and causes accountable for modeling output. The machine-learning technique and the input data determine how well a model can be interpreted. Non-neural network approaches typically contain fewer learnable parameters and feature sets that are more accessible to meaningful interpretation, making interpretation easier. For example, in a simple linear regression model, the parameter allotted to every input feature indicates how that variable influences the prediction. Because non-neural network approaches are inexpensive to train, ablation research in which the impact of eliminating certain input features on performance is quantified is recommended. One approach to potentially finding more reliable, effective, and understandable models is through ablation experiments, which can highlight which aspects are most helpful for a particular modeling job. Because a neural network often has many input parameters and features, interpreting one is often significantly more difficult.

### 7.4 Challenges in transdisciplinary partnerships

The main concern for data-driven crop improvement and production programs is standardized data collection protocol to prevent noisy data and the availability of high-resolution data. On the other hand, it is uncommon for one research organization to be aware of specific resources and knowledge to collect data in machine-learning research and adequately employ the most suitable machine-learning algorithms unless publicly available data are being used. Computer scientists and experimental agri-institutes frequently collaborate, and the outcomes of these collaborations are often outstanding. However, in these kinds of partnerships, each party must understand the other. In particular, agri-institutes and researchers should be aware of the constraints of the machine-learning algorithms being applied, and computer scientists should understand thoroughly the nature of the data, including the anticipated repeatability and level of noise. Developing such awareness takes time and work, but it is crucial for halting the frequent accidental spread of below-standard models and false conclusions.

## 8 Federated learning and gossip learning as recommendation approaches for global crop improvement and production programs

**Figure 7:**
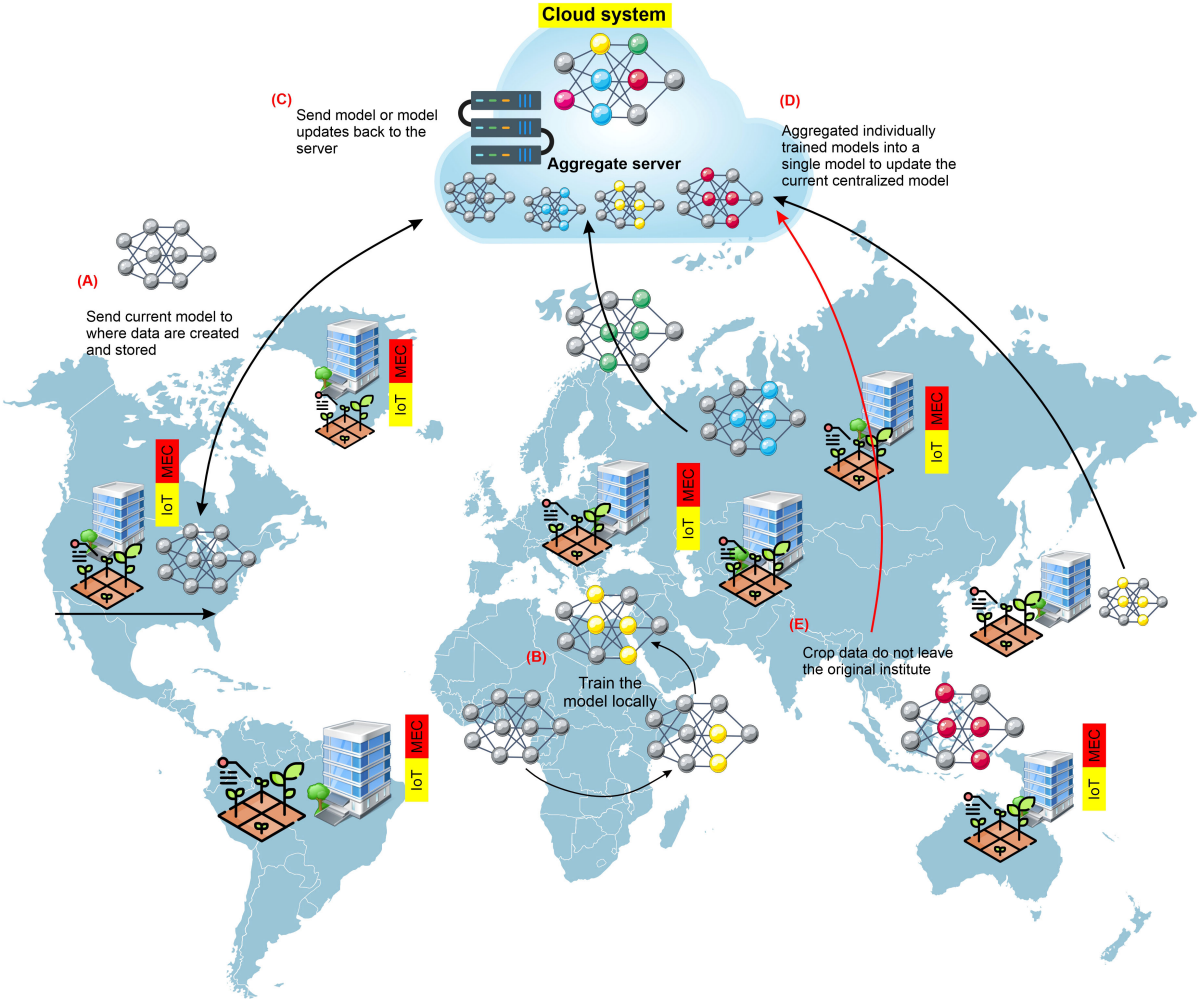
General pipeline constructing-silo for crop-improvement data to use in FL approaches. Several universities or institutes cooperatively train an ML model via federated learning (FL). In phase **(A)**, the central server provides the institution with the most recent model version. In phase **(B)**, each organization uses its data to train the model locally. In phase **(C)**, each institution transmits its trained model to the central server. In phase **(D)**, the central server combines all of the models that have been locally trained by the various universities into a single updated model. In phase **(E)**, each training cycle involves repeating this procedure till the training of the model is complete. Crop data never leave the institution during any of the training phases. Institutions need access to essential resources such as powerful hardware and specialists to conduct FL successfully.

When leveraging datasets from many institutions, the model could be trained centrally, combining data from silos of various institutions onto a single server. However, different legal, ethical, and administrative restrictions exist on publicly exchanging crop-based data. In many countries, crop-based data must remain in the group, company, or institution. Machine-learning models are trained using a decentralized method called federated learning, often called collaborative learning. Federated learning (FL) is an approach for building machine-learning models where distributed data are used cooperatively by a central server (McMahan et al., 2017; Kairouz et al., 2021), as illustrated in **Figure 7**. FL allows the data to remain at the original site to protect the safety and intellectual privacy of data, in contrast to centralized training, which transfers data from produced locations to a central server to train the model. Once a new training cycle begins, the most recent version of the model is transmitted to every storage site where the training data are stored (Greener et al., 2022). Each copy of the model is then trained and updated using the data that belong to each unique site. The revised models are then returned to the central server from each site, where they are merged to create a universal model. After that, the freshly revised universal model is released for distribution once more, and the cycle continues until either the model training or convergence is completed. Only those people directly related to that institution have direct access to the data, which means that the data are never virtually transported from the originating location or institution. In an FL approach, the risks of data ownership violations are decreased, data aggregation costs are kept to a minimum, and training datasets can quickly increase in size and variety. Optimum use of the FL approach can lay the groundwork for training deep-learning models for universal crop-based data.

### 8.1 FL taxonomy

**Figure 8:**
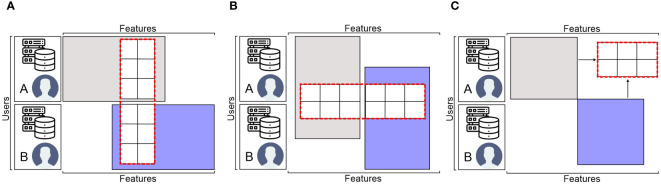
The FL data partition categories: **(A)** horizontal FL, **(B)** vertical FL, and **(C)** federated transfer learning.

The data matrix is the foundation of FL (Li et al., 2022). FL is categorized into three groups according to the various distribution patterns of the sample space and feature space of the data: federated transfer learning (FTL), vertical FL (VFL), and horizontal FL (HFL), which partition datasets non-dimensionally, longitudinally (i.e., dimension of features), and horizontally (i.e., dimension of users), correspondingly, as shown in **Figure 8**.

### 8.2 General workflow for employing the FL approach

Data holders and central servers are the usual components of FL systems (Li et al., 2022). Not enough local data or feature counts from individual data holders may be available to enable effective model training. As a result, cooperation from other data owners is needed. The FL procedure for the architecture of the client-server is shown in **Figure 7**. To safeguard data privacy, the data holders exclusively train their data locally in a standard cooperative modeling procedure of FL. After desensitization, the gradients produced by the iterations are used as interaction information and sent to a trustworthy third-party server in place of local data and, to update the model, the server should return the aggregated parameters. The stages involved in FL can be summed up in detail below. The first step is system initialization. In this step, the central server sends out the modeling work and tries to engage with the client. Local calculation is the second step. Upon opening the joint modeling job and initializing the system settings, it will be necessary for each data owner (holder) to initially carry out local measurements and calculations based on the data locally. Eventually, the third step is central polymerization. The central server compiles the estimated values after obtaining the computation results from various data owners (holders). Security, privacy, efficiency, and other concerns are considered and checked during the aggregation process in this step. Significantly, the FL central server’s functioning is comparable to that of a distributed machine-learning server, which gathers each data holder’s gradient and then produces a new gradient via server aggregation processes.

### 8.3 FL applications in agriculture and relevant work in some crops

Since FL allows datasets to be analyzed even when the raw data are either not readily available or the data owners are not ready to share target data, this opens tremendous opportunities to use the mentioned approach in different domains. In the medical field, FL has been used to recognize COVID-19 disease during the pandemic through an image analysis approach from chest-computed tomography (Lai and Yan, 2022). According to their findings, their network’s communication cost decreased by using the federated averaging model. Additionally, to lessen Byzantine assaults in their federated learning test bed, researchers suggest a modified federated learning model in which the edge nodes are randomly split into groups, each assigned a separate transmission time slot (Sifaou and Li, 2022). Because edge devices have a wide range of capabilities and resources, researchers have developed a federated learning framework that analyzes the models without jeopardizing data security or privacy while reaching convergence (Kevin et al., 2022). Agri-researchers, agri-institutes, and agri-companies also frequently gather private data and information that they prefer to keep private, as presented in **Table 3**. FL uses machine learning to train a shared model across several devices without requiring data exchange. It is perfect for agricultural applications. The FL applications in agriculture are categorized below to create a global model based on data-partitioning techniques, architecture, aggregation algorithms, and scale of federation. In one effort, researchers use a horizontally distributed dataset placed on several client devices to train the yield prediction model using FL (Manoj et al., 2022). To demonstrate the efficacy of agricultural data under decentralized learning, the FedAvg algorithm is used to build deep regression models such as ResNet-16. In another effort, to classify crops (chickpea, maize, and rice), the federated averaging approach has been employed (Idoje et al., 2023). Compared to the stochastic gradient descent (SGD) optimizer, the Adam optimizer model converged more quickly in this research. The study using the farm dataset has shown that decentralized models outperform centralized network models in terms of accuracy and convergence speed.

**Table 3:** Agricultural applications of the federated learning method in some crops.

Target area in agriculture	Issue	Number of customers	Challenges	Data used	Aggregation approaches	Trained model	Ref
Smart farming and crop classification	Data security in intelligent farming	6	Usage of FL in intelligent agriculture	The dataset included rainfall, pH, humidity, and temperature of independent variables	Model of federated averaging	CNN	(Idoje et al., 2023)
Production from the agricultural sector	Directing the production of agriculture	10	Inexpensive transmission, quick convergence rate, and precise modeling with limited resources	Soybean iron deficiency chlorosis (IDC) photos from the real world	A greedy algorithm and suggested a collaborative FL framework for the Edge-IoAT (Internet of Agriculture Things) framework to identify the best course of action	GA (greedy algorithm)	(Yu et al., 2022a)
Detection of various pests and diseases	To prevent imbalanced and inadequate orchard data, expensive data storage and transmission, various pests and diseases, and challenging detection situations for typical cloud-based deep-learning solutions	6	Prevent the communication costs that arise from uploading a lot of data to address the problem of imbalanced and inadequate data	445 images of orchard apples, of which only 152 images include five diseases	FedAvg approach	Improved faster region convolutional neural network (R-CNN)	(Deng et al., 2022)
Using FL for amendable multi-function control method for smart sensors for enhanced agricultural production	Enhancing efficiency	47	FL is derived from sensor information	Soil and crop data	Amendable multi-function sensor control method (AMFSC)	AMFSC	(Abu-Khadrah et al., 2023)
Disease detection in food crops	Anticipating leaf diseases	4	Privacy of data	Data from plant-village	FedAvg approach	Five CNNs: ShuffleNet, SqueezeNet, AlexNet, VGG-11, and ResNet-18	(Antico et al., 2022)

### 8.4 Federated learning challenges and limitation

Like other systems, FL also has some limitation and challenges for users which can categorized in four main groups include high-cost communication, heterogeneity of systems, heterogeneity in statistics, and privacy issues (Mammen, 2021; Moshawrab et al., 2023). The first challenges are raised in FL system is high-cost communication. Network communication in federated systems can be many orders of magnitude slower than local computing because these models consist of a large number computing devices. Compared to traditional data center facilities, communication in these networks can be substantially more expensive. It is also required to design communication-efficient approaches that iteratively send short messages or model updates as part of the training process, instead of sending the complete dataset over the network, in order to fit a model to data supplied by the devices in a federated network. The second challenge is heterogeneity of systems. Due to variations in hardware (memory, CPU), power, and network connectivity, each device in federated networks may have different computing, storage, and communication capabilities. Furthermore, only a small percentage of the devices are usually active at any given time due to the scale of the network and limits imposed by individual systems on each device. For instance, in a network with millions of devices, only hundreds of devices might be in use. It is also possible for any device to be unreliable, and it happens frequently for an active device to stop working during a particular cycle. Problems like stragglers and fault tolerance are far more common due to these system-level features than they are in standard data center settings. The third challenge od FL system is heterogeneity in statistics in the system. Across the FL network, devices typically produce and gather data in non-identically dispersed ways. Furthermore, there may be a large variation in the quantity of data points amongst devices. And finally, the last challenge of FL system is privacy concerns. In contrast to learning in data centers, privacy is frequently a primary problem in FL systems. FL only shares model updates rather than raw data, which is a step in the right direction towards preserving user data. Sensitive and important information may still be revealed to the central server or a third party by sharing model changes during the training phase. Although there have been efforts recently to improve FL privacy through the use of techniques like differential privacy or secure multiparty computing, these strategies frequently sacrifice system efficiency or model performance in order to achieve privacy (Zhang et al., 2021a; Wen et al., 2023).

## 9 Gossip learning can be alternative to federated learning

To tackle the same issue, gossip learning has also been suggested as an alternative to federated learning (Ormándi et al., 2013; Hegedűs et al., 2016, 2019). There is no need for a parameter server because this method is completely decentralized. Nodes immediately share and combine models. Undoubtedly, there are seveal advantages to using gossip learning because there is no single point of failure and gossip learning has far cheaper scalability and better resilience because no infrastructure is needed. The term “gossip” describes the information-sharing process that occurs over the network in a manner akin to that of gossip within a social group. In this approach, through information sharing with other nodes in the network, each node in the network updates its model parameters in this distributed machine learning technique. The theory is that any node can rapidly converge to the global optimum by exchanging information with other nodes. In large-scale distributed systems where node-to-node communication is unreliable or expensive, gossip learning is very helpful.

## 10 Conclusions and future direction

Future predictions display significantly greater use of AI and ML approaches in crop science, which could open a new horizon for integrated and valuable solutions in this area. We have undertaken a thorough review of the essential elements, concepts, applications, and machine-learning definitions required for agri-crop improvement. Nowadays, crop science is leveraging tons of available data to obtain deeper insights through AI and ML and offer the best suggestions for following actions and decisions for enhancing crop productivity or for other necessary tasks. Crop improvement and forecasting are made more accessible by combining computer science and agriculture. Offering broad recommendations and guidance for machine learning in agriculture is challenging because of the diversity of agricultural data. Therefore, our article aimed to provide agricultural and crop science researchers with an overview of the many accessible approaches, as well as some suggestions for conducting efficient machine learning through available data. It is vital to recognize that machine learning is inappropriate for all problems and to know when to avoid it: when the available data are insufficient, when it is necessary to comprehend rather than anticipate, or when it is not apparent how to fairly evaluate performance. Also, here we highlighted the application of federated learning in agriculture along with the definition, procedures, and structure, which can be beneficial for researchers in the agricultural sector. Even though there has been huge progress in machine learning in agriculture, many challenges still need to be addressed to mark ML territory in agricultural science. There is no denying that machine learning has influenced and will continue to influence agricultural research significantly.

## Author contributions

SMHK: Writing – review & editing, Writing – original draft, Visualization, Validation, Methodology, Investigation, Formal analysis, Data curation, Conceptualization. JA: Writing – review & editing, Supervision, Resources, Project administration, Methodology, Investigation, Funding acquisition, Conceptualization.

## References

[B1] Abadi M. Barham P. Chen J. Chen Z. Davis A. Dean J.. (2016) {TensorFlow}: a system for {Large-Scale} machine learning, 12th USENIX symposium on operating systems design and implementation (OSDI 16), 265–283.

[B2] Abdel-Nasser M. Mahmoud K. (2019). Accurate photovoltaic power forecasting models using deep LSTM-RNN. Neural computing Appl. 31, 2727–2740. doi: 10.1007/s00521-017-3225-z

[B3] Abidi A. Ienco D. Abbes A. B. Farah I. R. (2023). Combining 2D encoding and convolutional neural network to enhance land cover mapping from Satellite Image Time Series. Eng. Appl. Artif. Intell. 122, 106152. doi: 10.1016/j.engappai.2023.106152

[B4] Abinaya S. Devi M. K. (2022). Enhancing crop productivity through autoencoder-based disease detection and context-aware remedy recommendation system. Appl. Mach. Learn. Agric. (Cambridge, MA, USA: Academic Press), 239–262. doi: 10.1016/B978-0-323-90550-3.00014-X

[B5] Abu-Khadrah A. Ali A. M. Jarrah M. (2023). An amendable multi-function control method using federated learning for smart sensors in agricultural production improvements. ACM Trans. Sensor Networks. doi: 10.1145/3582011

[B6] Agarwal D. (2024). A machine learning framework for the identification of crops and weeds based on shape curvature and texture properties. Int. J. Inf. Technol. 16, 1261–1274. doi: 10.1007/s41870-023-01598-9

[B7] Aguilar-Zambrano J. Mambuscay C. A. A. Jaramillo-Botero A. (2023). *Omics sciences in agriculture: crop phenomes and microbiomes* Sello Editorial Javeriano-Pontificia Universidad Javeriana, Cali.

[B8] Ahfock D. McLachlan G. J. (2023). Semi-supervised learning of classifiers from a statistical perspective: A brief review. Econometrics Stat 26, 124–138. doi: 10.1016/j.ecosta.2022.03.007

[B9] Ahmed F. Al-Mamun H. A. Bari A. H. Hossain E. Kwan P. (2012). Classification of crops and weeds from digital images: A support vector machine approach. Crop Prot. 40, 98–104. doi: 10.1016/j.cropro.2012.04.024

[B10] Ahmed S. (2023). A software framework for predicting the maize yield using modified multi-layer perceptron. Sustainability 15, 3017. doi: 10.3390/su15043017

[B11] Ahmed U. Mumtaz R. Anwar H. Shah A. A. Irfan R. García-Nieto J. (2019). Efficient water quality prediction using supervised machine learning. Water 11, 2210. doi: 10.3390/w11112210

[B12] Akiba T. Sano S. Yanase T. Ohta T. Koyama M. (2019). “Optuna: A next-generation hyperparameter optimization framework,” in Proceedings of the 25th ACM SIGKDD international conference on knowledge discovery & data mining. Anchorage. doi: 10.1145/3292500.3330701

[B13] Alif A. A. Shukanya I. F. Afee T. N. (2018). Crop prediction based on geographical and climatic data using machine learning and deep learning (BRAC University).

[B14] Alzubaidi L. Bai J. Al-Sabaawi A. Santamaría J. Albahri A. S. Al-dabbagh B. S. N.. (2023). A survey on deep learning tools dealing with data scarcity: definitions, challenges, solutions, tips, and applications. J. Big Data 10, 46. doi: 10.1186/s40537-023-00727-2

[B15] Antico T. M. Moreira L. F. R. Moreira R. (2022). “Evaluating the potential of federated learning for maize leaf disease prediction,” in Anais do XIX Encontro Nacional de Inteligência Artificial e Computacional (SBC). doi: 10.5753/eniac.2022

[B16] Attri I. Awasthi L. K. Sharma T. P. (2024). Machine learning in agriculture: a review of crop management applications. Multimedia Tools Appl. 83, 12875–12915. doi: 10.1007/s11042-023-16105-2

[B17] Ayesha Barvin P. Sampradeepraj T. (2023). Crop recommendation systems based on soil and environmental factors using graph convolution neural network: A systematic literature review. Eng. Proc. 58, 97. doi: 10.3390/ecsa-10-16010

[B18] Bah M. D. Hafiane A. Canals R. (2023). Hierarchical graph representation for unsupervised crop row detection in images. Expert Syst. Appl. 216, 119478. doi: 10.1016/j.eswa.2022.119478

[B19] Bank D. Koenigstein N. Giryes R. (2023). Autoencoders. Machine Learning for Data Science Handbook: Data Mining and Knowledge Discovery Handbook (NY, USA: Springer New York).

[B20] Basha S. M. Rajput D. S. Janet J. Somula R. S. Ram S. (2020). Principles and practices of making agriculture sustainable: crop yield prediction using Random Forest. Scalable Computing: Pract. Exp. 21, 591–599. doi: 10.12694/scpe.v21i4.1714

[B21] Battaglia P. W. Hamrick J. B. Bapst V. Sanchez-Gonzalez A. Zambaldi V. Malinowski M.. (2018). Relational inductive biases, deep learning, and graph networks. arXiv preprint. arXiv:1806.01261. doi: 10.48550/arXiv.1806.01261

[B22] Baxter J. (2000). A model of inductive bias learning. J. Artif. Intell. Res. 12, 149–198. doi: 10.1613/jair.731

[B23] Bengio Y. (2012). Practical recommendations for gradient-based training of deep architectures, *Neural networks: Tricks of the trade: Second edition* (Springer), 437–478.

[B24] Ben-Hur A. Ong C. S. Sonnenburg S. Schölkopf B. Rätsch G. (2008). Support vector machines and kernels for computational biology. PloS Comput. Biol. 4, e1000173. doi: 10.1371/journal.pcbi.1000173 18974822 PMC2547983

[B25] Ben-Hur A. Weston J. (2010). “A user’s guide to support vector machines,” in Data mining techniques for the life sciences, 223–239. 10.1007/978-1-60327-241-4_13 20221922

[B26] Bergstra J. Komer B. Eliasmith C. Yamins D. Cox D. D. (2015). Hyperopt: a python library for model selection and hyperparameter optimization. Comput. Sci. Discovery 8, 014008. doi: 10.1088/1749-4699/8/1/014008

[B27] Bhosle K. Musande V. (2022). Evaluation of CNN model by comparing with convolutional autoencoder and deep neural network for crop classification on hyperspectral imagery. Geocarto Int. 37, 813–827. doi: 10.1080/10106049.2020.1740950

[B28] Bian K. Priyadarshi R. (2024). Machine learning optimization techniques: a Survey, classification, challenges, and Future Research Issues. Arch. Comput. Methods Eng., 1–25. doi: 10.1007/s11831-024-10110-w

[B29] Blaom A. D. Kiraly F. Lienart T. Simillides Y. Arenas D. Vollmer S. J. (2020). MLJ: A Julia package for composable machine learning. arXiv preprint arXiv:2007.12285. doi: 10.48550/arXiv.2007.12285

[B30] Bouguettaya A. Zarzour H. Kechida A. Taberkit A. M. (2023). A survey on deep learning-based identification of plant and crop diseases from UAV-based aerial images. Cluster Computing 26, 1297–1317. doi: 10.1007/s10586-022-03627-x 35968221 PMC9362359

[B31] Boukhris A. Jilali A. Asri H. (2024). Deep learning and machine learning based method for crop disease detection and identification using autoencoder and neural network. Rev. d’Intelligence Artificielle 38, 459–472. doi: 10.18280/ria

[B32] Bouvrie J. (2006). Notes on convolutional neural networks.

[B33] Brahim J. Loubna R. Noureddine F. (2021). RNN-and CNN-based weed detection for crop improvement: An overview. Foods Raw materials 9, 387–396. doi: 10.21603/2308-4057-2021-2-387-396

[B34] Butte S. Vakanski A. Duellman K. Wang H. Mirkouei A. (2021). Potato crop stress identification in aerial images using deep learning-based object detection. Agron. J. 113, 3991–4002. doi: 10.1002/agj2.20841

[B35] Bzdok D. Krzywinski M. Altman N. (2018). Machine learning: supervised methods. Nat. Methods 15, 5. doi: 10.1038/nmeth.4551 30100821 PMC6082635

[B36] Cao J. Zhang Z. Tao F. Zhang L. Luo Y. Zhang J.. (2021). Integrating multi-source data for rice yield prediction across China using machine learning and deep learning approaches. Agric. For. Meteorology 297, 108275. doi: 10.1016/j.agrformet.2020.108275

[B37] Chang H.-X. Haudenshield J. S. Bowen C. R. Hartman G. L. (2017). Metagenome-wide association study and machine learning prediction of bulk soil microbiome and crop productivity. Front. Microbiol. 8, 519. doi: 10.3389/fmicb.2017.00519 28421041 PMC5378059

[B38] Chatterjee T. Gogoi U. R. Samanta A. Chatterjee A. Singh M. K. Pasupuleti S. (2024). Identifying the most discriminative parameter for water quality prediction using machine learning algorithms. Water 16, 481. doi: 10.3390/w16030481

[B39] Chen T. Guestrin C. (2016). “Xgboost: A scalable tree boosting system,” in Proceedings of the 22nd acm sigkdd international conference on knowledge discovery and data mining. doi: 10.1145/2939672

[B40] Chen Y. Huang Y. Zhang Z. Wang Z. Liu B. Liu C.. (2023). Plant image recognition with deep learning: A review. Comput. Electron. Agric. 212, 108072. doi: 10.1016/j.compag.2023.108072

[B41] Chrysostomou C. Seker H. Aydin N. (2011). “Effects of windowing and zero-padding on complex resonant recognition model for protein sequence analysis,” in 2011 Annual International Conference of the IEEE Engineering in Medicine and Biology Society, Boston, MA, USA. doi: 10.1109/IEMBS.2011.6091228 22255450

[B42] Colmer J. O’Neill C. M. Wells R. Bostrom A. Reynolds D. Websdale D.. (2020). SeedGerm: a cost-effective phenotyping platform for automated seed imaging and machine-learning based phenotypic analysis of crop seed germination. New Phytol. 228, 778–793. doi: 10.1111/nph.16736 32533857

[B43] Crick F. (1989). The recent excitement about neural networks. Nature 337, 129–132. doi: 10.1038/337129a0 2911347

[B44] Cui S. Su Y. L. Duan K. Liu Y. (2023). Maize leaf disease classification using CBAM and lightweight Autoencoder network. J. Ambient Intell. Humanized Computing 14, 7297–7307. doi: 10.1007/s12652-022-04438-z

[B45] Dahouda M. K. Joe I. (2021). A deep-learned embedding technique for categorical features encoding. IEEE Access 9, 114381–114391. doi: 10.1109/ACCESS.2021.3104357

[B46] Danilevicz M. F. Gill M. Anderson R. Batley J. Bennamoun M. Bayer P. E.. (2022). Plant genotype to phenotype prediction using machine learning. Front. Genet. 13, 822173. doi: 10.3389/fgene.2022.822173 35664329 PMC9159391

[B47] Das P. Ivkin N. Bansal T. Rouesnel L. Gautier P. Karnin Z.. (2020). “Amazon SageMaker Autopilot: a white box AutoML solution at scale,” in Proceedings of the fourth international workshop on data management for end-to-end machine learning. doi: 10.1145/3399579

[B48] Davis R. L. Greene J. K. Dou F. Jo Y.-K. Chappell T. M. (2020). A practical application of unsupervised machine learning for analyzing plant image data collected using unmanned aircraft systems. Agronomy 10, 633. doi: 10.3390/agronomy10050633

[B49] Dege D. Brüggemann P. (2023). Marketing analytics with RStudio: a software review (Springer). doi: 10.1057/s41270-023-00264-0

[B50] Deng F. Mao W. Zeng Z. Zeng H. Wei B. (2022). Multiple diseases and pests detection based on federated learning and improved faster R-CNN. IEEE Trans. Instrumentation Measurement 71, 1–11. doi: 10.1109/TIM.2022.3201937

[B51] Dhaliwal D. S. Williams M. M. (2024). Sweet corn yield prediction using machine learning models and field-level data. Precis. Agric. 25, 51–64. doi: 10.1007/s11119-023-10057-1

[B52] Dhillon M. S. Dahms T. Kuebert-Flock C. Rummler T. Arnault J. Steffan-Dewenter I.. (2023). Integrating random forest and crop modeling improves the crop yield prediction of winter wheat and oil seed rape. Front. Remote Sens. 3, 1010978. doi: 10.3389/frsen.2022.1010978

[B53] Dhillon R. Takoo G. Sharma V. Nagle M. (2024). Utilizing machine learning framework to evaluate the effect of climate change on maize and soybean yield. Comput. Electron. Agric. 221, 108982. doi: 10.1016/j.compag.2024.108982

[B54] Diao Z. Yan J. He Z. Zhao S. Guo P. (2022). Corn seedling recognition algorithm based on hyperspectral image and lightweight-3D-CNN. Comput. Electron. Agric. 201, 107343. doi: 10.1016/j.compag.2022.107343

[B55] Doersch C. (2016). Tutorial on variational autoencoders. arXiv preprint. arXiv:1606.05908. doi: 10.48550/arXiv.1606.05908

[B56] Du C. Jiang S. Chen C. Guo Q. He Q. Zhan C. (2024). Machine learning-based estimation of daily cropland evapotranspiration in diverse climate zones. Remote Sens. 16, 730. doi: 10.3390/rs16050730

[B57] Du Z. Yang L. Zhang D. Cui T. He X. Xiao T.. (2022). Corn variable-rate seeding decision based on gradient boosting decision tree model. Comput. Electron. Agric. 198, 107025. doi: 10.1016/j.compag.2022.107025

[B58] Duc N. T. Ramlal A. Rajendran A. Raju D. Lal S. Kumar S.. (2023). Image-based phenotyping of seed architectural traits and prediction of seed weight using machine learning models in soybean. Front. Plant Sci. 14, 1206357. doi: 10.3389/fpls.2023.1206357 37771485 PMC10523016

[B59] Durrani A. U. R. Minallah N. Aziz N. Frnda J. Khan W. Nedoma J. (2023). Effect of hyper-parameters on the performance of ConvLSTM based deep neural network in crop classification. PloS One 18, e0275653. doi: 10.1371/journal.pone.0275653 36758037 PMC9910738

[B60] Eelbode T. Sinonquel P. Maes F. Bisschops R. (2021). Pitfalls in training and validation of deep learning systems. Best Pract. Res. Clin. Gastroenterol. 52, 101712. doi: 10.1016/j.bpg.2020.101712 34172245

[B61] Elango E. Hanees A. Shanmuganathan B. Kareem Basha M. I. (2024). “Precision Agriculture: A Novel Approach on AI-Driven Farming,” in Intelligent Robots and Drones for Precision Agriculture (Springer), 119–137.

[B62] Elavarasan D. Vincent P. D. (2020). Crop yield prediction using deep reinforcement learning model for sustainable agrarian applications. IEEE Access 8, 86886–86901. doi: 10.1109/Access.6287639

[B63] Elbasi E. Zaki C. Topcu A. E. Abdelbaki W. Zreikat A. I. Cina E.. (2023). Crop prediction model using machine learning algorithms. Appl. Sci. 13, 9288. doi: 10.3390/app13169288

[B64] Ester M. Kriegel H.-P. Sander J. Xu X. (1996). A density-based algorithm for discovering clusters in large spatial databases with noise. 2nd International Conference on Knowledge Discovery and Data Mining (KDD-96). kdd., 226–23.

[B65] Evamoni F. Z. Nulit R. Yap C. K. Ibrahim M. H. Sidek N. B. (2023). Assessment of germination performance and early seedling growth of Malaysian indica rice genotypes under drought conditions for strategic cropping during water scarcity. Chilean J. Agric. Res. 83, 281–292. doi: 10.4067/S0718-58392023000300281

[B66] Fey M. Lenssen J. E. (2019). Fast graph representation learning with PyTorch Geometric. arXiv preprint arXiv:1903.02428. doi: 10.48550/arXiv.1903.02428

[B67] Fu X. Ma Q. Yang F. Zhang C. Zhao X. Chang F.. (2023). Crop pest image recognition based on the improved ViT method. Inf. Process. Agric. 11 (2), 249–259. 10.1016/j.inpa.2023.02.007

[B68] Gafurov A. Mukharamova S. Saveliev A. Yermolaev O. (2023). Advancing agricultural crop recognition: the application of LSTM networks and spatial generalization in satellite data analysis. Agriculture 13, 1672. doi: 10.3390/agriculture13091672

[B69] Gano B. Bhadra S. Vilbig J. M. Ahmed N. Sagan V. Shakoor N. (2024). Drone-based imaging sensors, techniques, and applications in plant phenotyping for crop breeding: A comprehensive review. Plant Phenome J. 7, e20100. doi: 10.1002/ppj2.20100

[B70] Gao H. Ji S. (2019). “Graph u-nets,” in International conference on machine learning.

[B71] Gauriau O. Galárraga L. Brun F. Termier A. Davadan L. Joudelat F. (2024). Comparing machine-learning models of different levels of complexity for crop protection: A look into the complexity-accuracy tradeoff. Smart Agric. Technol. 7, 100380. doi: 10.1016/j.atech.2023.100380

[B72] Ge W. Zhou J. Zheng P. Yuan L. Rottok L. T. (2024). A recommendation model of rice fertilization using knowledge graph and case-based reasoning. Comput. Electron. Agric. 219, 108751. doi: 10.1016/j.compag.2024.108751

[B73] Gholap P. S. Sharma G. Deepak A. Madan P. Sharma R. Sharma M.. (2024). IoT enabled stress detection based on image processing with ensembling machine learning approach. Int. J. Intelligent Syst. Appl. Eng. 12, 760–768.

[B74] Ghosal A. Nandy A. Das A. K. Goswami S. Panday M. (2020). “A short review on different clustering techniques and their applications,” in Emerging Technology in Modelling and Graphics: Proceedings of IEM Graph, Vol. 2018. 69–83.

[B75] Ghosh H. Tusher M. A. Rahat I. S. Khasim S. Mohanty S. N. (2023). “Water quality assessment through predictive machine learning,” in International Conference on Intelligent Computing and Networking.

[B76] Goodfellow I. Bengio Y. Courville A. (2016). Deep learning (MIT press).

[B77] Gopi P. Karthikeyan M. (2024). Red fox optimization with ensemble recurrent neural network for crop recommendation and yield prediction model. Multimedia Tools Appl. 83, 13159–13179. doi: 10.1007/s11042-023-16113-2

[B78] Greener J. G. Kandathil S. M. Moffat L. Jones D. T. (2022). A guide to machine learning for biologists. Nat. Rev. Mol. Cell Biol. 23, 40–55. doi: 10.1038/s41580-021-00407-0 34518686

[B79] Guo J. Li H. Ning J. Han W. Zhang W. Zhou Z.-S. (2020). Feature dimension reduction using stacked sparse auto-encoders for crop classification with multi-temporal, quad-pol SAR Data. Remote Sens. 12, 321. doi: 10.3390/rs12020321

[B80] Guo T. Li X. (2023). Machine learning for predicting phenotype from genotype and environment. Curr. Opin. Biotechnol. 79, 102853. doi: 10.1016/j.copbio.2022.102853 36463837

[B81] Hamidi M. Homayouni S. Safari A. Hasani H. (2024). Deep learning based crop-type mapping using SAR and optical data fusion. Int. J. Appl. Earth Observation Geoinformation 129, 103860. doi: 10.1016/j.jag.2024.103860

[B82] Hamidi M. Safari A. Homayouni S. (2021). An auto-encoder based classifier for crop mapping from multitemporal multispectral imagery. Int. J. Remote Sens. 42, 986–1016. doi: 10.1080/01431161.2020.1820619

[B83] Hara K. Kataoka H. Satoh Y. (2018). “Can spatiotemporal 3d cnns retrace the history of 2d cnns and imagenet?,” in Proceedings of the IEEE conference on Computer Vision and Pattern Recognition. doi: 10.1109/CVPR.2018.00685

[B84] Hastie T. Tibshirani R. Friedman J. H. Friedman J. H. (2009). *The elements of statistical learning: data mining, inference, and prediction*, *2* (Springer). doi: 10.1007/978-0-387-84858-7

[B85] Heffner E. L. Sorrells M. E. Jannink J. L. (2009). Genomic selection for crop improvement. Crop Sci. 49, 1–12. doi: 10.2135/cropsci2008.08.0512

[B86] Hegedűs I. Berta Á. Kocsis L. Benczúr A. A. Jelasity M. (2016). Robust decentralized low-rank matrix decomposition. ACM Trans. Intelligent Syst. Technol. (TIST) 7, 1–24. doi: 10.1145/2854157

[B87] Hegedűs I. Danner G. Jelasity M. (2019). “Gossip learning as a decentralized alternative to federated learning,” in Distributed Applications and Interoperable Systems.

[B88] Herrera J. M. Häner L. L. Holzkämper A. Pellet D. (2018). Evaluation of ridge regression for country-wide prediction of genotype-specific grain yields of wheat. Agric. For. meteorology 252, 1–9. doi: 10.1016/j.agrformet.2017.12.263

[B89] Ho Y. Wookey S. (2019). The real-world-weight cross-entropy loss function: Modeling the costs of mislabeling. IEEE Access 8, 4806–4813. doi: 10.1109/Access.6287639

[B90] Hu K. Wang Z. Coleman G. Bender A. Yao T. Zeng S.. (2021). Deep learning techniques for in-crop weed identification: A review. arXiv preprint arXiv:2103.14872. doi: 10.1007/s11119-023-10073-1

[B91] Huber F. Yushchenko A. Stratmann B. Steinhage V. (2022). Extreme Gradient Boosting for yield estimation compared with Deep Learning approaches. Comput. Electron. Agric. 202, 107346. doi: 10.1016/j.compag.2022.107346

[B92] Idoje G. Dagiuklas T. Iqbal M. (2023). Federated Learning: Crop classification in a smart farm decentralised network. Smart Agric. Technol. 5, 100277. doi: 10.1016/j.atech.2023.100277

[B93] Iniyan S. Varma V. A. Naidu C. T. (2023). Crop yield prediction using machine learning techniques. Adv. Eng. Software 175, 103326. doi: 10.1016/j.advengsoft.2022.103326

[B94] Jain A. K. (2010). Data clustering: 50 years beyond K-means. Pattern recognition Lett. 31, 651–666. doi: 10.1016/j.patrec.2009.09.011

[B95] Jain T. Garg P. Tiwari P. K. Kuncham V. K. Sharma M. Verma V. K. (2021). “Performance prediction for crop irrigation using different machine learning approaches,” in Examining the Impact of Deep Learning and IoT on Multi-Industry Applications (IGI Global), 61–79.

[B96] James G. Witten D. Hastie T. Tibshirani R. Taylor J. (2023). “Unsupervised learning,” in An Introduction to Statistical Learning: with Applications in Python (Springer), 503–556. doi: 10.1007/978-3-031-38747-0

[B97] Jeong J. H. Resop J. P. Mueller N. D. Fleisher D. H. Yun K. Butler E. E.. (2016). Random forests for global and regional crop yield predictions. PloS One 11, e0156571. doi: 10.1371/journal.pone.0156571 27257967 PMC4892571

[B98] Ji S. Zhang C. Xu A. Shi Y. Duan Y. (2018). 3D convolutional neural networks for crop classification with multi-temporal remote sensing images. Remote Sens. 10, 75. doi: 10.3390/rs10010075

[B99] Jiang Y. Li C. (2020). Convolutional neural networks for image-based high-throughput plant phenotyping: a review. Plant Phenomics. doi: 10.34133/2020/4152816 PMC7706326 33313554

[B100] Jiang H. Zhang C. Qiao Y. Zhang Z. Zhang W. Song C. (2020). CNN feature based graph convolutional network for weed and crop recognition in smart farming. Comput. Electron. Agric. 174, 105450. doi: 10.1016/j.compag.2020.105450

[B101] Jolliffe I. T. Cadima J. (2016). Principal component analysis: a review and recent developments. Philos. Trans. R. Soc. A: Mathematical Phys. Eng. Sci. 374, 20150202. doi: 10.1098/rsta.2015.0202 PMC4792409 26953178

[B102] Kairouz P. McMahan H. B. Avent B. Bellet A. Bennis M. Bhagoji A. N.. (2021). Advances and open problems in federated learning. Foundations trends® Mach. Learn. 14, 1–210. doi: 10.1561/2200000083

[B103] Kamatchi C. B. Muthukumaravel A. (2024). “Machine learning in agriculture: A land data approach to optimize crop choice with the LAGNet model,” in 2024 International Conference on Emerging Smart Computing and Informatics (ESCI), Pune, India. doi: 10.1109/ESCI59607.2024.10497261

[B104] Kashyap G. R. Sridhara S. Manoj K. N. Gopakkali P. Das B. Jha P. K.. (2024). Machine learning ensembles, neural network, hybrid and sparse regression approaches for weather based rainfed cotton yield forecast. Int. J. Biometeorol. 68, 1179–1197. doi: 10.1007/s00484-024-02661-1 38676745

[B105] Kavitha E. Jadhav H. M. Goyal V. Deepak A. Pokhariya H. S. Sharma B. D.. (2024). Utilizing convolutional neural networks for image-based crop classification system. Int. J. Intelligent Syst. Appl. Eng. 12, 685–694.

[B106] Kevin I. Wang K. Ye X. Sakurai K. (2022). “Federated learning with clustering-based participant selection for IoT applications,” in 2022 IEEE International Conference on Big Data (Big Data). Osaka, Japan. doi: 10.1109/BigData55660.2022.10020575

[B107] Kheir A. Nangia V. Elnashar A. Devakota M. Omar M. Feike T.. (2024). Developing automated machine learning approach for fast and robust crop yield prediction using a fusion of remote sensing, soil, and weather dataset. Environ. Res. Commun. doi: 10.1088/2515-7620/ad2d02

[B108] Khoshnevisan B. Bolandnazar E. Barak S. Shamshirband S. Maghsoudlou H. Altameem T. A.. (2015). A clustering model based on an evolutionary algorithm for better energy use in crop production. Stochastic Environ. Res. Risk Assess. 29, 1921–1935. doi: 10.1007/s00477-014-0972-6

[B109] Killeen P. Kiringa I. Yeap T. Branco P. (2024). Corn grain yield prediction using UAV-based high spatiotemporal resolution imagery, machine learning, and spatial cross-validation. Remote Sens. 16, 683. doi: 10.3390/rs16040683

[B110] Kircher M. Witten D. M. Jain P. O’roak B. J. Cooper G. M. Shendure J. (2014). A general framework for estimating the relative pathogenicity of human genetic variants. Nat. Genet. 46, 310–315. doi: 10.1038/ng.2892 24487276 PMC3992975

[B111] Kolipaka V. R. R. Namburu A. (2024). An automatic crop yield prediction framework designed with two-stage classifiers: a meta-heuristic approach. Multimedia Tools Appl. 83, 28969–28992. doi: 10.1007/s11042-023-16612-2

[B112] Kondermann D. (2013). “Ground truth design principles: an overview,” in Proceedings of the International Workshop on Video and Image Ground Truth in Computer Vision Applications. ACM: New York, NY, USA. doi: 10.1145/2501105

[B113] Korani W. Clevenger J. P. Chu Y. Ozias-Akins P. (2019). Machine learning as an effective method for identifying true single nucleotide polymorphisms in polyploid plants. Plant Genome 12, 180023. doi: 10.3835/plantgenome2018.05.0023 PMC12962348 30951095

[B114] Kuhn M. (2008). Building predictive models in R using the caret package. J. Stat. software 28, 1–26. doi: 10.18637/jss.v028.i05

[B115] Kulkarni P. Shastri S. (2024). Rice leaf diseases detection using machine learning. J. Sci. Res. Technol., 17–22. doi: 10.61808/jsrt81

[B116] Lai W. Yan Q. (2022). “Federated learning for detecting COVID-19 in chest CT images: a lightweight federated learning approach,” in 2022 4th International Conference on Frontiers Technology of Information and Computer (ICFTIC). Qingdao, China. doi: 10.1109/ICFTIC57696.2022.10075165

[B117] LeCun Y. Bengio Y. Hinton G. (2015). Deep learning. Nature 521, 436–444. doi: 10.1038/nature14539 26017442

[B118] Li D. Han D. Weng T.-H. Zheng Z. Li H. Liu H.. (2022). Blockchain for federated learning toward secure distributed machine learning systems: a systemic survey. Soft Computing 26, 4423–4440. doi: 10.1007/s00500-021-06496-5 34840525 PMC8605788

[B119] Li Z. Huffman T. Zhang A. Zhou F. McConkey B. (2012). Spatially locating soil classes within complex soil polygons–Mapping soil capability for agriculture in Saskatchewan Canada. Agriculture Ecosyst. Environ. 152, 59–67. doi: 10.1016/j.agee.2012.02.007

[B120] Li Z. Liu F. Yang W. Peng S. Zhou J. (2021). A survey of convolutional neural networks: analysis, applications, and prospects. IEEE Trans. Neural Networks Learn. Syst. 33, 6999–7019. doi: 10.1109/TNNLS.2021.3084827 34111009

[B121] Li L. Zhang Y. Wang B. Feng P. He Q. Shi Y.. (2023). Integrating machine learning and environmental variables to constrain uncertainty in crop yield change projections under climate change. Eur. J. Agron. 149, 126917. doi: 10.1016/j.eja.2023.126917

[B122] Liakos K. G. Busato P. Moshou D. Pearson S. Bochtis D. (2018). Machine learning in agriculture: A review. Sensors 18, 2674. doi: 10.3390/s18082674 30110960 PMC6111295

[B123] Liao C. Wang J. Xie Q. Baz A. A. Huang X. Shang J.. (2020). Synergistic use of multi-temporal RADARSAT-2 and VENµS data for crop classification based on 1D convolutional neural network. Remote Sens. 12, 832. doi: 10.3390/rs12050832

[B124] Lingwal S. Bhatia K. K. Singh M. (2024). A novel machine learning approach for rice yield estimation. J. Exp. Theor. Artif. Intell. 36, 337–356. doi: 10.1080/0952813X.2022.2062458

[B125] Liu J. Wang T. Skidmore A. Sun Y. Jia P. Zhang K. (2023). Integrated 1D, 2D, and 3D CNNs enable robust and efficient land cover classification from hyperspectral imagery. Remote Sens. 15, 4797. doi: 10.3390/rs15194797

[B126] Liu T. Zhai D. He F. Yu J. (2024). Semi-supervised learning methods for weed detection in turf. Pest Manage. Sci. doi: 10.1002/ps.7959 38265105

[B127] Madala K. Prasad M. S. G. (2023). Crop mapping through hybrid capsule transient auto-encoder technique based on radar features. Multimedia Tools Appl. 8, 1–31. doi: 10.1007/s11042-023-17327-0

[B128] Mammen P. M. (2021). Federated learning: Opportunities and challenges. arXiv preprint arXiv:2101.05428. doi: 10.48550/arXiv.2101.05428

[B129] Manoj T. Makkithaya K. Narendra V. (2022). “A federated learning-based crop yield prediction for agricultural production risk management,” in 2022 IEEE Delhi Section Conference (DELCON). New Delhi, India. doi: 10.1109/DELCON54057.2022.9752836.

[B130] Masjedi A. Carpenter N. R. Crawford M. M. Tuinstra M. R. (2019). “Prediction of sorghum biomass using UAV time series data and recurrent neural networks,” in Proceedings of the IEEE/CVF Conference on Computer Vision and Pattern Recognition Workshops. doi: 10.1109/CVPRW47913.2019

[B131] Mathai N. Chen Y. Kirchmair J. (2020). Validation strategies for target prediction methods. Briefings Bioinf. 21, 791–802. doi: 10.1093/bib/bbz026 PMC7299289 31220208

[B132] Mazzia V. Khaliq A. Chiaberge M. (2019). Improvement in land cover and crop classification based on temporal features learning from Sentinel-2 data using recurrent-convolutional neural network (R-CNN). Appl. Sci. 10, 238. doi: 10.3390/app10010238

[B133] McMahan B. Moore E. Ramage D. Hampson S. Arcas B. (2017). “Communication-efficient learning of deep networks from decentralized data,” in Artificial intelligence and statistics. Seattle, WA 98103 USA.

[B134] Medsker L. R. Jain L. (2001). Recurrent neural networks. Design Appl. 5, 2.

[B135] Meenal R. Jala P. K. Samundeswari R. Rajasekaran E. (2024). Crop water management using machine learning-based evapotranspiration estimation. J. Appl. Biol. Biotechnol. 12, 198–203. doi: 10.7324/JABB.2024.155791

[B136] Memon R. Memon M. Malioto N. Raza M. O. (2021). “Identification of growth stages of crops using mobile phone images and machine learning,” in 2021 International conference on computing, Electronic and Electrical Engineering (ICE Cube). Quetta, Pakistan. doi: 10.1109/ICECube53880.2021.9628197

[B137] Mochida K. Koda S. Inoue K. Hirayama T. Tanaka S. Nishii R.. (2019). Computer vision-based phenotyping for improvement of plant productivity: a machine learning perspective. GigaScience 8, giy153. doi: 10.1093/gigascience/giy153 30520975 PMC6312910

[B138] Modi R. U. Kancheti M. Subeesh A. Raj C. Singh A. K. Chandel N. S.. (2023). An automated weed identification framework for sugarcane crop: a deep learning approach. Crop Prot. 173, 106360. doi: 10.1016/j.cropro.2023.106360

[B139] Moharram M. A. Sundaram D. M. (2023). Land Use and Land Cover Classification with Hyperspectral Data: A comprehensive review of methods, challenges and future directions. Neurocomputing. doi: 10.1016/j.neucom.2023.03.025

[B140] Morales A. Villalobos F. J. (2023). Using machine learning for crop yield prediction in the past or the future. Front. Plant Sci. 14, 1128388. doi: 10.3389/fpls.2023.1128388 37063228 PMC10097960

[B141] Mora-Poblete F. Maldonado C. Henrique L. Uhdre R. Scapim C. A. Mangolim C. A. (2023). Multi-trait and multi-environment genomic prediction for flowering traits in maize: a deep learning approach. Front. Plant Sci. 14, 1153040. doi: 10.3389/fpls.2023.1153040 37593046 PMC10428628

[B142] Mosavi A. Samadianfard S. Darbandi S. Nabipour N. Qasem S. N. Salwana E.. (2021). Predicting soil electrical conductivity using multi-layer perceptron integrated with grey wolf optimizer. J. Geochemical Explor. 220, 106639. doi: 10.1016/j.gexplo.2020.106639

[B143] Moshawrab M. Adda M. Bouzouane A. Ibrahim H. Raad A. (2023). Reviewing federated learning aggregation algorithms; strategies, contributions, limitations and future perspectives. Electronics 12, 2287. doi: 10.3390/electronics12102287

[B144] Mousavi S. R. Jahandideh Mahjenabadi V. A. Khoshru B. Rezaei M. (2024). Spatial prediction of winter wheat yield gap: agro-climatic model and machine learning approaches. Front. Plant Sci. 14, 1309171. doi: 10.3389/fpls.2023.1309171 38264030 PMC10804884

[B145] Nar K. Ocal O. Sastry S. S. Ramchandran K. (2019). Cross-entropy loss and low-rank features have responsibility for adversarial examples. arXiv preprint arXiv:1901.08360. doi: 10.48550/arXiv.1901.08360

[B146] Neal B. (2019). On the bias-variance tradeoff: Textbooks need an update. arXiv preprint arXiv:1912.08286. doi: 10.48550/arXiv.1912.08286

[B147] Nejad S. M. M. Abbasi-Moghadam D. Sharifi A. Farmonov N. Amankulova K. Lászlź M. (2022). Multispectral crop yield prediction using 3D-convolutional neural networks and attention convolutional LSTM approaches. IEEE J. Selected Topics Appl. Earth Observations Remote Sens. 16, 254–266. doi: 10.1109/JSTARS.2022.3223423

[B148] Nevavuori P. Narra N. Lipping T. (2019). Crop yield prediction with deep convolutional neural networks. Comput. Electron. Agric. 163, 104859. doi: 10.1016/j.compag.2019.104859

[B149] Noble W. S. (2006). What is a support vector machine? Nat. Biotechnol. 24, 1565–1567. doi: 10.1038/nbt1206-1565 17160063

[B150] Olson R. S. Cava W. L. Mustahsan Z. Varik A. Moore J. H. (2018). “Data-driven advice for applying machine learning to bioinformatics problems,” in Pacific Symposium on Biocomputing 2018: Proceedings of the Pacific Symposium. doi: 10.1142/10864 PMC5890912 29218881

[B151] Opach T. Rød J. K. (2018). Augmenting the usability of parallel coordinate plot: The polyline glyphs. Inf. Visualization 17, 108–127. doi: 10.1177/1473871617693041

[B152] Ormándi R. Hegedűs I. Jelasity M. (2013). Gossip learning with linear models on fully distributed data. Concurrency Computation: Pract. Exp. 25, 556–571. doi: 10.48550/arXiv.1109.1396

[B153] Osman Y. Dennis R. Elgazzar K. (2021). Yield estimation and visualization solution for precision agriculture. Sensors 21, 6657. doi: 10.3390/s21196657 34640977 PMC8512698

[B154] Ouali Y. Hudelot C. Tami M. (2020). An overview of deep semi-supervised learning. arXiv preprint arXiv:2006.05278. doi: 10.48550/arXiv.2006.05278

[B155] Pandey S. Yadav P. K. Sahu R. Pandey P. (2024). “Improving crop management with convolutional neural networks for binary and multiclass weed recognition,” in 2024 2nd International Conference on Intelligent Data Communication Technologies and Internet of Things (IDCIoT). Bengaluru, India. doi: 10.1109/IDCIoT59759.2024.10467501

[B156] Panigrahi B. Kathala K. C. R. Sujatha M. (2023). A machine learning-based comparative approach to predict the crop yield using supervised learning with regression models. Proc. Comput. Sci. 218, 2684–2693. doi: 10.1016/j.procs.2023.01.241

[B157] Pardoe I. (2020). Applied regression modeling (John Wiley & Sons). doi: 10.1002/9781119615941

[B158] Paszke A. Gross S. Massa F. Lerer A. Bradbury J. Chanan G.. (2019). Pytorch: An imperative style, high-performance deep learning library. Adv. Neural Inf. Process. Syst. 32, 8026–8037. doi: 10.48550/arXiv.1912.01703

[B159] Pedregosa F. Varoquaux G. Gramfort A. Michel V. Thirion B. Grisel O.. (2011). Scikit-learn: machine learning in python. J. Mach. Learn. Res. 12, 2825–2830.

[B160] Peterson L. E. (2009). K-nearest neighbor. Scholarpedia 4, 1883. doi: 10.4249/scholarpedia.1883

[B161] Picon A. San-Emeterio M. G. Bereciartua-Perez A. Klukas C. Eggers T. Navarra-Mestre R. (2022). Deep learning-based segmentation of multiple species of weeds and corn crop using synthetic and real image datasets. Comput. Electron. Agric. 194, 106719. doi: 10.1016/j.compag.2022.106719

[B162] Piekutowska M. Niedbała G. Piskier T. Lenartowicz T. Pilarski K. Wojciechowski T.. (2021). The application of multiple linear regression and artificial neural network models for yield prediction of very early potato cultivars before harvest. Agronomy 11, 885. doi: 10.3390/agronomy11050885

[B163] Rajamani S. K. Iyer R. S. (2023). “Machine Learning-Based Mobile Applications Using Python and Scikit-Learn,” in Designing and developing innovative mobile applications (IGI Global), 282–306.

[B164] Rangarajan A. K. Purushothaman R. Prabhakar M. Szczepański C. (2023). Crop identification and disease classification using traditional machine learning and deep learning approaches. J. Eng. Res. 11, 228–252. doi: 10.36909/jer.11941

[B165] Rodríguez P. Bautista M. A. Gonzalez J. Escalera S. (2018). Beyond one-hot encoding: Lower dimensional target embedding. Image Vision Computing 75, 21–31. doi: 10.1016/j.imavis.2018.04.004

[B166] Sahoo R. N. Rejith R. Gakhar S. Ranjan R. Meena M. C. Dey A.. (2024). Drone remote sensing of wheat N using hyperspectral sensor and machine learning. Precis. Agric. 25, 704–728. doi: 10.1007/s11119-023-10089-7

[B167] Sarkar C. Gupta D. Gupta U. Hazarika B. B. (2023). Leaf disease detection using machine learning and deep learning: Review and challenges. Appl. Soft Computing 22, 110534. doi: 10.1016/j.asoc.2023.110534

[B168] Sehrawat S. Najafian K. Jin L. (2023). Predicting phenotypes from novel genomic markers using deep learning. Bioinf. Adv. 3, vbad028. doi: 10.1093/bioadv/vbad028 PMC10132579 37123455

[B169] Seising R. (2018). The emergence of fuzzy sets in the decade of the perceptron—Lotfi A. Zadeh’s and frank rosenblatt’s research work on pattern classification. Mathematics 6, 110.

[B170] Sejnowski T. J. (2018). The deep learning revolution (MIT press). doi: 10.7551/mitpress/11474.001.0001

[B171] Sen P. C. Hajra M. Ghosh M. (2020). “Supervised classification algorithms in machine learning: A survey and review,” in Emerging Technology in Modelling and Graphics: Proceedings of IEM Graph 2018.

[B172] Shafiee S. Lied L. M. Burud I. Dieseth J. A. Alsheikh M. Lillemo M. (2021). Sequential forward selection and support vector regression in comparison to LASSO regression for spring wheat yield prediction based on UAV imagery. Comput. Electron. Agric. 183, 106036. doi: 10.1016/j.compag.2021.106036

[B173] Sharma A. Jain A. Gupta P. Chowdary V. (2020). Machine learning applications for precision agriculture: A comprehensive review. IEEE Access 9, 4843–4873. doi: 10.1109/Access.6287639

[B174] Shin A. Kim D. Y. Jeong J. S. Chun B.-G. (2020). Hippo: Taming hyperparameter optimization of deep learning with stage trees. arXiv preprint arXiv:2006.11972.

[B175] Sifaou H. Li G. Y. (2022). “Robust federated learning via over-the-air computation,” in 2022 IEEE 32nd International Workshop on Machine Learning for Signal Processing (MLSP). doi: 10.1109/MLSP55214.2022.9943401

[B176] Sindhu Meena K. Suriya S. (2020). “A survey on supervised and unsupervised learning techniques,” in Proceedings of international conference on artificial intelligence, smart grid and smart city applications: AISGSC 2019.

[B177] Smith A. M. Walsh J. R. Long J. Davis C. B. Henstock P. Hodge M. R.. (2020). Standard machine learning approaches outperform deep representation learning on phenotype prediction from transcriptomics data. BMC Bioinf. 21, 1–18. doi: 10.1186/s12859-020-3427-8 PMC7085143 32197580

[B178] Srinivas L. Bharathy A. V. Ramakuri S. K. Sethy A. Kumar R. (2024). An optimized machine learning framework for crop disease detection. Multimedia Tools Appl. 83, 1539–1558. doi: 10.1007/s11042-023-15446-2

[B179] Su X. Yan X. Tsai C. L. (2012). Linear regression. Wiley Interdiscip. Reviews: Comput. Stat 4, 275–294.

[B180] Sudha M. K. Manorama M. Aditi T. (2022). Smart agricultural decision support systems for predicting soil nutrition value using IoT and ridge regression. AGRIS on-line Papers Economics Inf. 14, 95–106. doi: 10.7160/aol.2022.140108

[B181] Sun Z. Di L. Fang H. (2019). Using long short-term memory recurrent neural network in land cover classification on Landsat and Cropland data layer time series. Int. J. Remote Sens. 40, 593–614. doi: 10.1080/01431161.2018.1516313

[B182] Tang Z. Hu Y. Zhang Z. (2023). ROOSTER: An image labeler and classifier through interactive recurrent annotation. F1000Research 12, 137. doi: 10.12688/f1000research

[B183] Tang W. Long G. Liu L. Zhou T. Jiang J. Blumenstein M. (2020). Rethinking 1d-cnn for time series classification: A stronger baseline. arXiv preprint arXiv:2002.10061, 1–7.

[B184] Tian Y. Yang C. Huang W. Tang J. Li X. Zhang Q. (2021). Machine learning-based crop recognition from aerial remote sensing imagery. Front. Earth Sci. 15, 54–69. doi: 10.1007/s11707-020-0861-x

[B185] Tibshirani R. (1996). Regression shrinkage and selection via the lasso. J. R. Stat. Soc. Ser. B: Stat. Method. 58, 267–288. doi: 10.1111/j.2517-6161.1996.tb02080.x

[B186] Tong H. Nikoloski Z. (2021). Machine learning approaches for crop improvement: Leveraging phenotypic and genotypic big data. J. Plant Physiol. 257, 153354. doi: 10.1016/j.jplph.2020.153354 33385619

[B187] Twomey J. M. Smith A. E. (1997). Validation and verification. Artif. Neural Networks civil engineers: Fundamentals Appl. (New York: ASCE), 44–64.

[B188] Uma A. N. Fornaciari T. Hovy D. Paun S. Plank B. Poesio M. (2021). Learning from disagreement: A survey. J. Artif. Intell. Res. 72, 1385–1470. doi: 10.1613/jair.1.12752

[B189] Uppu S. Krishna A. Gopalan R. P. (2016). A deep learning approach to detect SNP interactions. J. Softw 11, 965–975. doi: 10.17706/jsw.11.10.965-975

[B190] Van der Maaten L. Hinton G. (2008). Visualizing data using t-SNE. J. Mach. Learn. Res. 9.

[B191] Vani P. S. Rathi S. (2023). Improved data clustering methods and integrated A-FP algorithm for crop yield prediction. Distributed Parallel Database 41, 117–131.

[B192] Van Klompenburg T. Kassahun A. Catal C. (2020). Crop yield prediction using machine learning: A systematic literature review. Comput. Electron. Agric. 177, 105709. doi: 10.1016/j.compag.2020.105709

[B193] Veenadhari S. Misra B. Singh C. (2014). “Machine learning approach for forecasting crop yield based on climatic parameters,” in 2014 International Conference on Computer Communication and Informatics. doi: 10.1109/ICCCI.2014.6921718

[B194] Venkataraju A. Arumugam D. Stepan C. Kiran R. Peters T. (2023). A review of machine learning techniques for identifying weeds in corn. Smart Agric. Technol. 3, 100102. doi: 10.1016/j.atech.2022.100102

[B195] Wang C. Zhang Y. (2017). Improving scoring-docking-screening powers of protein–ligand scoring functions using random forest. J. Comput. Chem. 38, 169–177. doi: 10.1002/jcc.24667 27859414 PMC5140681

[B196] Wei Q. Dunbrack R. L. Jr. (2013). The role of balanced training and testing data sets for binary classifiers in bioinformatics. PloS One 8, e67863. doi: 10.1371/journal.pone.0067863 23874456 PMC3706434

[B197] Wei J. Zhang M. Wu C. Ma Q. Wang W. Wan C. (2024). Accurate crop row recognition of maize at the seedling stage using lightweight network. Int. J. Agric. Biol. Eng. 17, 189–198. doi: 10.25165/j.ijabe.20241701.7051

[B198] Wen J. Zhang Z. Lan Y. Cui Z. Cai J. Zhang W. (2023). A survey on federated learning: challenges and applications. Int. J. Mach. Learn. Cybernetics 14, 513–535. doi: 10.1007/s13042-022-01647-y PMC9650178 36407495

[B199] Wu Y.-C. Feng J.-W. (2018). Development and application of artificial neural network. Wireless Pers. Commun. 102, 1645–1656. doi: 10.1007/s11277-017-5224-x

[B200] Wu D. Yang Z. Li T. Liu J. (2024). JOCP: A jointly optimized clustering protocol for industrial wireless sensor networks using double-layer selection evolutionary algorithm. Concurrency Computation: Pract. Exp. 36, e7927.

[B201] Yamaç S. S. Todorovic M. (2020). Estimation of daily potato crop evapotranspiration using three different machine learning algorithms and four scenarios of available meteorological data. Agric. Water Manage. 228, 105875. doi: 10.1016/j.agwat.2019.105875

[B202] Yang F. Zhang D. Zhang Y. Zhang Y. Han Y. Zhang Q.. (2023). Prediction of corn variety yield with attribute-missing data via graph neural network. Comput. Electron. Agric. 211, 108046. doi: 10.1016/j.compag.2023.108046

[B203] Yesugade K. Kharde A. Mirashi K. Muley K. Chudasama H. (2018). Machine learning approach for crop selection based on agro-climatic conditions. Mach. Learn. 7. doi: 10.17148/IJARCCE

[B204] Yoosefzadeh-Najafabadi M. Eskandari M. Torabi S. Torkamaneh D. Tulpan D. Rajcan I. (2022). Machine-learning-based genome-wide association studies for uncovering QTL underlying soybean yield and its components. Int. J. Mol. Sci. 23, 5538. doi: 10.3390/ijms23105538 35628351 PMC9141736

[B205] Yu C. Shen S. Zhang K. Zhao H. Shi Y. (2022a). “Energy-aware device scheduling for joint federated learning in edge-assisted internet of agriculture things,” in 2022 IEEE Wireless Communications and Networking Conference (WCNC). doi: 10.1109/WCNC51071.2022.9771547

[B206] Yu L. Zhou R. Chen R. Lai K. K. (2022b). Missing data preprocessing in credit classification: One-hot encoding or imputation? Emerging Markets Finance Trade 58, 472–482. doi: 10.1080/1540496X.2020.1825935

[B207] Yu T. Zhu H. (2020). Hyper-parameter optimization: A review of algorithms and applications. arXiv preprint arXiv:2003.05689.

[B208] Zanella M. A. Martins R. N. da Silva F. M. Carvalho L. C. C. de Carvalho Alves M. Rosas J. T. F. (2024). Coffee yield prediction using high-resolution satellite imagery and crop nutritional status in Southeast Brazil. Remote Sens. Applications: Soc. Environ. 33, 101092.

[B209] Zhang Z. Boubin J. Stewart C. Khanal S. (2020). Whole-field reinforcement learning: A fully autonomous aerial scouting method for precision agriculture. Sensors 20, 6585. doi: 10.3390/s20226585 33218000 PMC7698769

[B210] Zhang T. Cai Y. Zhuang P. Li J. (2024). Remotely sensed crop disease monitoring by machine learning algorithms: A review. Unmanned Syst. 12, 161–171. doi: 10.1142/S2301385024500237

[B211] Zhang C. Xie Y. Bai H. Yu B. Li W. Gao Y. (2021a). A survey on federated learning. Knowledge-Based Syst. 216, 106775. doi: 10.1016/j.knosys.2021.106775

[B212] Zhang L. Zhang Z. Tao F. Luo Y. Cao J. Li Z.. (2021b). Planning maize hybrids adaptation to future climate change by integrating crop modelling with machine learning. Environ. Res. Lett. 16, 124043. doi: 10.1088/1748-9326/ac32fd

[B213] Zou H. Hastie T. (2005). Regularization and variable selection via the elastic net. J. R. Stat. Soc. Ser. B: Stat. Method. 67, 301–320. doi: 10.1111/j.1467-9868.2005.00503.x

